# Europium‐ and dysprosium‐modified strontium aluminate: A luminescent marker system for enhanced gunshot residue detection

**DOI:** 10.1111/1556-4029.70328

**Published:** 2026-04-12

**Authors:** Paulo Tonin, Cesar Aguzzoli, Sidnei Moura

**Affiliations:** ^1^ Laboratory of Biotechnology of Natural and Synthetics Products University of Caxias do Sul Caxias do Sul Brazil; ^2^ Postgraduate Program in Materials Science and Engineering University of Caxias do Sul (UCS) Caxias do Sul Brazil

**Keywords:** chemical markers, forensic ballistics, forensic materials, gunshot residue analysis, photoluminescent markers, SEM‐EDS

## Abstract

Forensic ballistics is essential in analyzing evidence from firearm use in criminal investigations, with gunshot residue (GSR) detection providing key information about the firearm and shooting conditions. Traditional methods like spectroscopy and scanning electron microscopy (SEM) have limitations in sensitivity, cost, and accessibility. Luminescent markers, especially lanthanide‐doped aluminates, offer a promising alternative by enabling residue visualization under specific lighting without complex equipment. This study evaluates europium‐ and dysprosium‐doped strontium aluminate as a luminescent marker for GSR detection. The compound was characterized by SEM‐EDS, laser diffraction, X‐ray diffraction, and thermogravimetric analysis. Three concentrations (5%, 10%, and 15% w/w relative to the propellant mass) were tested, revealing an average particle size of 64.43 μm, monocyclic structure, and high thermal stability. SEM‐EDS confirmed marker presence on target surfaces, and UV illumination allowed clear residue visualization post‐discharge. The 15% concentration showed optimal adhesion and persistence. These results suggest europium‐ and dysprosium‐doped strontium aluminate as a promising tool for rapid and accurate GSR detection in forensic applications, complemented by a literature review on current GSR identification techniques.

**Highlights**

- Luminescent markers provide a rapid, nondestructive screening tool for GSR at crime scenes.
- SrAl_2_O_4_:Eu^3+^Dy^3+^ exhibited strong UV‐induced luminescence and high thermal stability up to ~900°C.
- Visual identification of residues under UV light enhances the efficiency of evidence collection.
- Doped strontium aluminate ensures chemical stability and persistence during firearm discharge.
- This system offers a scalable solution for marking ammunition to track forensic evidence.

## 1 INTRODUCTION

Gunshot residues (GSR) are formed following the rapid explosion that occurs with each firearm discharge. These residues emanate through openings in the cartridge case head after the propellant combustion, releasing gases containing both burned and unburned particles [1, 2]. In forensic investigations, these residues represent crucial evidence indicating firearm discharge, as every firearm produces GSR that disperses both in the environment and on the shooter [3, 4].

GSR can be classified into three main categories: inorganic gunshot residues (IGSR), organic gunshot residues (OGSR), and luminescent gunshot residues (LGSR) [5, 6]. The American Society for Testing and Materials established the ASTM E1588‐20 standard, Standard Practice for Gunshot Residue Analysis by Scanning Electron Microscopy/Energy Dispersive X‐Ray Spectrometry (SEM‐EDS), which serves as the standard protocol for GSR analysis, particularly for inorganic compounds [7]. In traditional forensic examinations, IGSR is identified by the presence of characteristic particles containing lead, barium, and antimony, typically showing spherical, fused, or agglomerated morphologies [8, 9, 10]. However, SEM‐EDS presents limitations: the technique is time‐consuming, requires specialized instrumentation and trained personnel, and its performance is compromised when characteristic metals are absent or reduced, as observed with modern nontoxic ammunition [1, 3]. We recently published a review highlighting key techniques beyond SEM‐EDS and X‐ray, addressing challenges in IGSR and OGSR characterization [11].

Currently, some ammunition types, known as nontoxic ammunition, lack these characteristic metals. These alternatives feature primer compounds replaced with less toxic substances and jacketed projectiles that prevent lead aerosol formation during discharge. While these ammunition types do not produce characteristic IGSR, complicating residue analysis [9, 12], they do generate characteristic OGSR during the combustion of propellants and additives. These additives include sensitizers, plasticizers, stabilizers, lubricants, coolants, and flash inhibitors, with over 180 compounds reported in the literature [5, 13, 14, 15]. Because IGSR and OGSR originate from different processes (primer combustion and propellant/additive decomposition, respectively), their detection provides independent but complementary information. For this reason, several authors recommend combining IGSR and OGSR analyses, as this approach increases the chance of detecting at least one residue type, improves case discrimination, and helps reduce false negatives, particularly when nontoxic ammunition or environmentally degraded samples are involved [5, 13, 14]. Additionally, OGSR compounds exhibit greater susceptibility to environmental degradation and shorter persistence at the crime scene compared to inorganic residues, due to their volatility and chemical instability, which further complicates detection in routine casework [5].

However, OGSR analysis lacks a standardized technique. Common analytical methods include Fourier transform infrared spectroscopy, various chromatographic techniques coupled with mass spectrometry, and other specialized analytical approaches [1, 11, 13, 15, 16]. This variability presents a challenge for forensic analysts. In an effort to address this issue, the Organization of Scientific Area Committees for Forensic Science (OSAC) has recently proposed a formal practice recommending the identification of OGSR compounds using Liquid Chromatography/Mass Spectrometry, representing an initial step toward future methodological standardization; however, this guideline remains under evaluation and has not yet been widely implemented in forensic laboratories [17]. Consequently, research has focused on incorporating marker compounds into ammunition based on three principles: environmental rarity, thermal stability during discharge with subsequent persistence, and ease of analysis, preferably through simple techniques like UV light detection [6, 18, 19].

The addition of luminescent markers has gained attention, simplifying shooter identification through UV light detection. This enables on‐site detection, facilitates collection, and allows for confirmatory analysis using other analytical techniques [6, 19]. Luminescent gunshot residue is visually detectable because the marker disperses across the firing area and on the shooter, and can be excited using inexpensive ultraviolet light sources, which greatly simplifies preliminary identification [6, 20]. Lanthanide‐containing compounds, particularly those incorporating europium, terbium, and dysprosium, are frequently reported in the literature [6, 21, 22, 23]. Recent studies have also demonstrated the use of lanthanide‐based metal–organic frameworks (LMOFs) as luminescent markers, as these materials combine high structural organization, thermal and chemical stability, and strong emission under UV excitation, making them promising candidates for ammunition additives [21, 23, 24]. This study aims to evaluate the applicability of europium and dysprosium‐doped strontium aluminate (SrAl_2_O_4_:Eu^3+^Dy^3+^) as a luminescent marker for detecting GSR. We present a comprehensive physicochemical characterization and stability testing of these compounds as ammunition additives.

## 2 MATERIALS AND METHODS

### 2.1 Luminescent marker

The luminescent marker, europium‐ and dysprosium‐doped strontium aluminate (SrAl_2_O_4_:Eu^3+^Dy^3+^), was commercially obtained from Luminstant (São Paulo, SP, Brazil). According to the supplier, the material is provided as a dry, nonencapsulated, water‐insoluble phosphor with a pale greenish‐white appearance, and no particle size information is disclosed. Before the experiments, the powder was dried in an oven at 100°C for 2 h and subsequently stored in a desiccator until the preparation of the ammunition, in order to minimize moisture‐related luminescence quenching. No additional treatments or processing steps were performed, and the material was used exactly as received, aside from the drying procedure. The compound underwent comprehensive physicochemical characterization and thermal stability assessment to investigate its behavior under high‐temperature conditions, simulating the internal thermal environment of a cartridge during discharge.

### 2.2 Thermogravimetric analysis (TGA)

TGA was performed using a Shimadzu TGA‐50 instrument (Kyoto, Japan). Approximately 12.39 mg of sample was placed in a platinum crucible and heated from room temperature to 910°C at a controlled heating rate of 10°C/min. The analysis was conducted under an inert atmosphere using nitrogen as the purge gas at a flow rate of 50 mL min^−1^, following parameters adapted from ASTM E1131 and Gültekin et al. [25].

### 2.3 High‐temperature treatment in a muffle furnace

Thermal degradation tests were conducted using a Quimis Q318D24 muffle furnace (São Paulo, Brazil) across multiple temperature ranges to evaluate the persistence of strontium aluminate luminescence under high‐temperature conditions and assess its viability for ammunition applications. Approximately 5 g of the material was placed in porcelain crucibles in triplicate and exposed to temperatures of 900, 1100, and 1300°C for 1 h. Temperature control was maintained by the equipment's thermocouple with a variation of ±5°C, following methodology adapted from Huang et al. [26]. Total solids content was determined using the following equation:

$$\text{Total solids}=\frac{\text{Mass of total solids}}{\text{Initial sample mass}}\times 100$$

### 2.4 Particle size distribution (PSD) by laser diffraction

PSD analysis was performed using a Fritsch Analysette 22 Nanotec Plus laser diffractometer (Germany) equipped with a dry dispersion system. The analysis was conducted at atmospheric pressure (1 atm) and ambient temperature (23 ± 2°C). Measurements were carried out in dry mode, covering a particle size range from 0.08 to 2000 μm. In this mode, sample amount control is not performed; instead, the sample is gradually added until the device signal meter detects an adequate level and initiates the measurement. No prior sample preparation was required. Three independent measurements were conducted, and data were processed using the Fraunhofer calculation method, chosen due to the irregular particle morphology.

### 2.5 Morphological analysis by SEM‐EDS

SEM‐EDS imaging was performed using a scanning electron microscope (Mira 3, Tescan, Czech Republic). The chemical composition was simultaneously verified with an EDS silicon drift detector (Oxford Instruments X‐act). Samples were mounted on aluminum stubs using carbon tape and coated with a thin gold layer to improve conductivity and image quality. Micrographs were acquired under vacuum with a 10 kV acceleration voltage, probe size 3, using the secondary electron (SE) mode. Elemental mapping was performed in qualitative mode using backscattered electrons (BSE) at 15 kV and a 15 mm working distance. In addition, SEM imaging was conducted on the propellant–marker mixtures prepared for ammunition reloading to document the morphology and dispersion of the different concentration ratios.

### 2.6 Structural characterization by X‐ray diffraction (XRD)

XRD analysis was performed using a Shimadzu XRD‐6000 diffractometer (Kyoto, Japan) equipped with Cu Kα radiation. Measurements were carried out at 40 kV and 30 mA, using a divergence slit of 1.0°, a scatter slit of 1.0°, and a receiving slit of 0.3 mm. Data were collected in continuous scan mode over a 2*θ* range of 5°–80°, with a scan speed of 1.2° min^−1^ and a sampling pitch of 0.02°, with a preset integration time of 1 s per step. In addition to the untreated material, XRD was also performed on the sample previously heated at 900°C to evaluate possible structural modifications after thermal exposure.

### 2.7 Fourier transform infrared (FTIR) spectroscopy

FTIR spectroscopy was performed using a PerkinElmer Spectrum 400 spectrometer in the ATR mode. A total of 30 scans were collected over the spectral range of 4000–450 cm^−1^ with a resolution of 4 cm^−1^. FTIR analysis was also conducted on the sample previously heated at 900°C to assess potential changes in vibrational modes after thermal exposure.

### 2.8 Firearm, ammunition, and shooting tests

Shooting tests were conducted in an enclosed range at the *Clube de Caça e Tiro Classe 5* in Caxias do Sul, RS, Brazil. The tests employed a Taurus® 0.38 MAG revolver with a 5.1‐in barrel and eight‐round capacity. Reloaded CBC cartridges were prepared using CBC primers, Morigi projectiles, and Fox single‐base powder. Following the methodology adapted from Lucena et al. [24], 15 cartridges were assembled using 0.65 g of powder and three different concentrations of the luminescent marker (5%, 10%, and 15% by mass), with five cartridges produced for each concentration. Three firing series were conducted, each consisting of five shots using cartridges containing one of the three marker concentrations. Different shooters performed the tests, alternating between series. Prior to each sequence, all participants thoroughly washed their hands with running water and neutral soap, following modified protocols from Lucena et al. [24].

### 2.9 Post‐discharge marker evaluation

Duplicate samples were collected using carbon tape for subsequent SEM‐EDS analysis, targeting specific hand regions: palm, back of the hand, thumb–palm grip, and thumb–back grip, in accordance with ASTM E1588‐20 and established forensic protocols for GSR collection. After each firing sequence, both the firearm and the shooters were visually examined under UV light to detect photoluminescent residues. Inspection was performed using a handheld UV‐B LED flashlight emitting at 254 nm (RZXLED UV‐B UVC LED Handheld Flashlight, Shenzhen, China), applied to the hands, arms, and clothing.

## 3 RESULTS AND DISCUSSION

The modernization of ammunition and growing concerns regarding environmental preservation and shooters' occupational health have created opportunities for nontoxic ammunition (NTA) to occupy a prominent position in the ammunition market. However, the absence of lead, barium, and antimony in their composition complicates GSR characterization. Consequently, there is a need to incorporate markers into NTA, facilitating simple and precise GSR detection [9, 12, 27]. In this context, luminescent markers play a crucial role in GSR detection by enabling rapid, on‐site identification. Such markers must possess specific characteristics: they should be cost‐effective, demonstrate good chemical stability to prevent reactions with ammunition components, maintain ballistic properties, and exhibit thermal stability to withstand the high temperatures generated during discharge. Additionally, these markers should contain compounds rarely found in nature to prevent confusion with molecules commonly present in work environments [21, 28].

Based on these requirements, we selected SrAl_2_O_4_:Eu^3+^Dy^3+^ to evaluate its viability as a luminescent marker, representing a novel application for this compound. This material is characterized by its stability, white appearance with a slight greenish tint, fine powder morphology, amorphous character, and absence of odor. When excited under UV light at 254 nm, the photoluminescent pigment emits a characteristic aqua‐blue luminescence. In addition, several studies report that this compound is nontoxic, nonradioactive, and environmentally benign [26, 29, 30].

### 3.1 Thermal stability assessment by TGA

**FIGURE 1:**
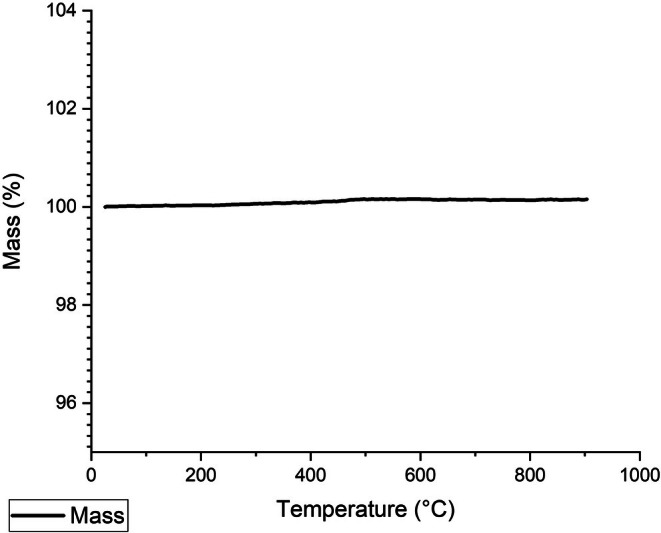
TGA curve showing the thermal behavior of the salt sample evaluated for ballistic additive applications. Analysis performed at 10°C/min from ambient temperature to 910°C.

To further characterize the compound's thermal behavior, thermogravimetric analysis was conducted. The TGA results (Figure 1) reveal exceptional thermal stability, with the material maintaining consistent mass up to approximately 910°C. A minimal mass increase of 0.34% was observed, likely resulting from residual moisture uptake, which is commonly reported for aluminates under heating. These observations correspond with findings reported by Zhu et al. [31], supporting the compound's stable thermal characteristics and resistance to mass loss at elevated temperatures.

**FIGURE 2:**
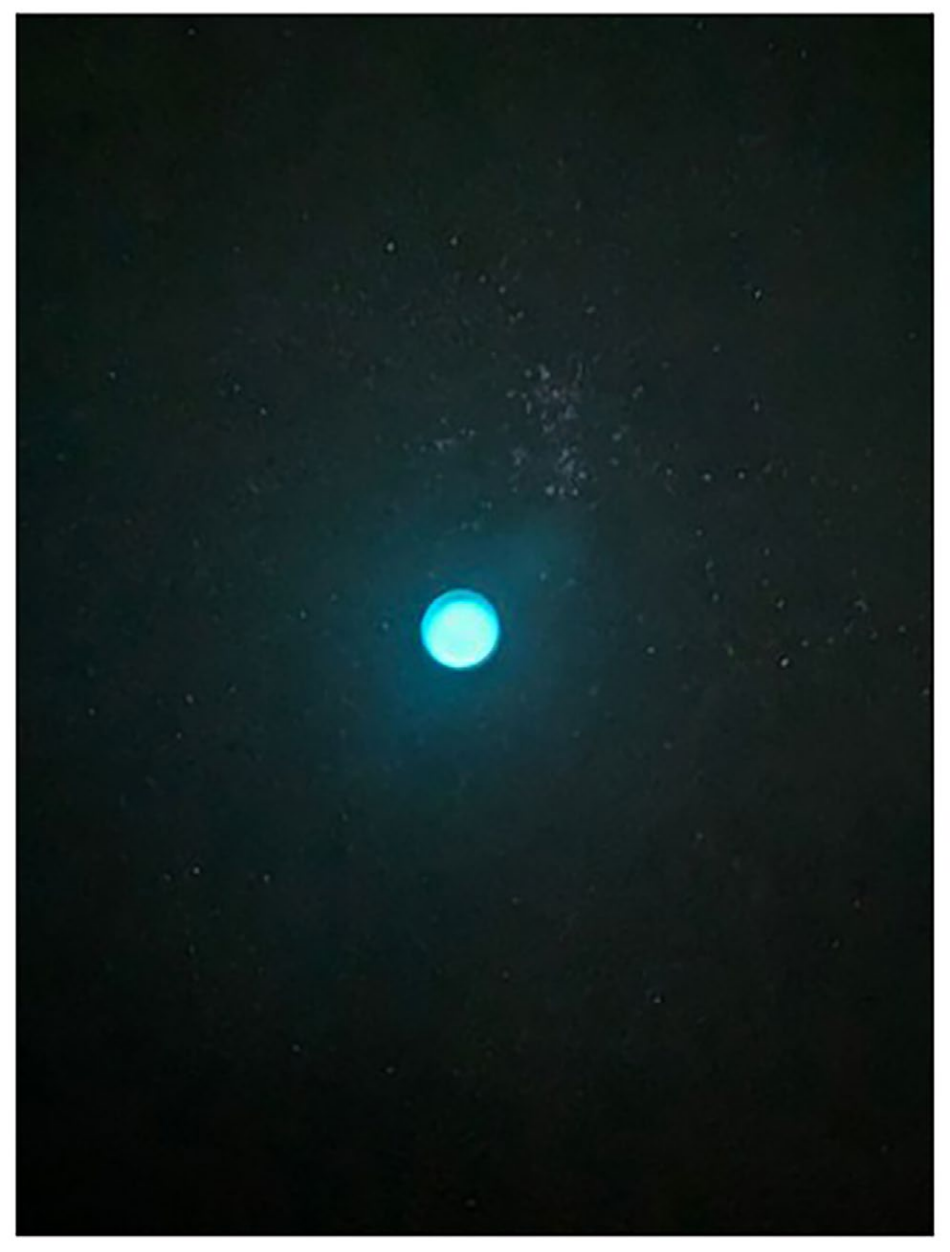
Sample luminescence under 254 nm UV light following thermal stability characterization by TGA, showing preserved photoluminescence characteristics.

The sample recovered after TGA was then examined under 254 nm UV radiation to confirm the preservation of its luminescent characteristics. As illustrated in Figure 2, the material continued to exhibit its characteristic luminescence, indicating that its photoluminescent properties remain stable even after exposure to high temperatures.

### 3.2 Thermal stability and total solids determination

**FIGURE 3:**
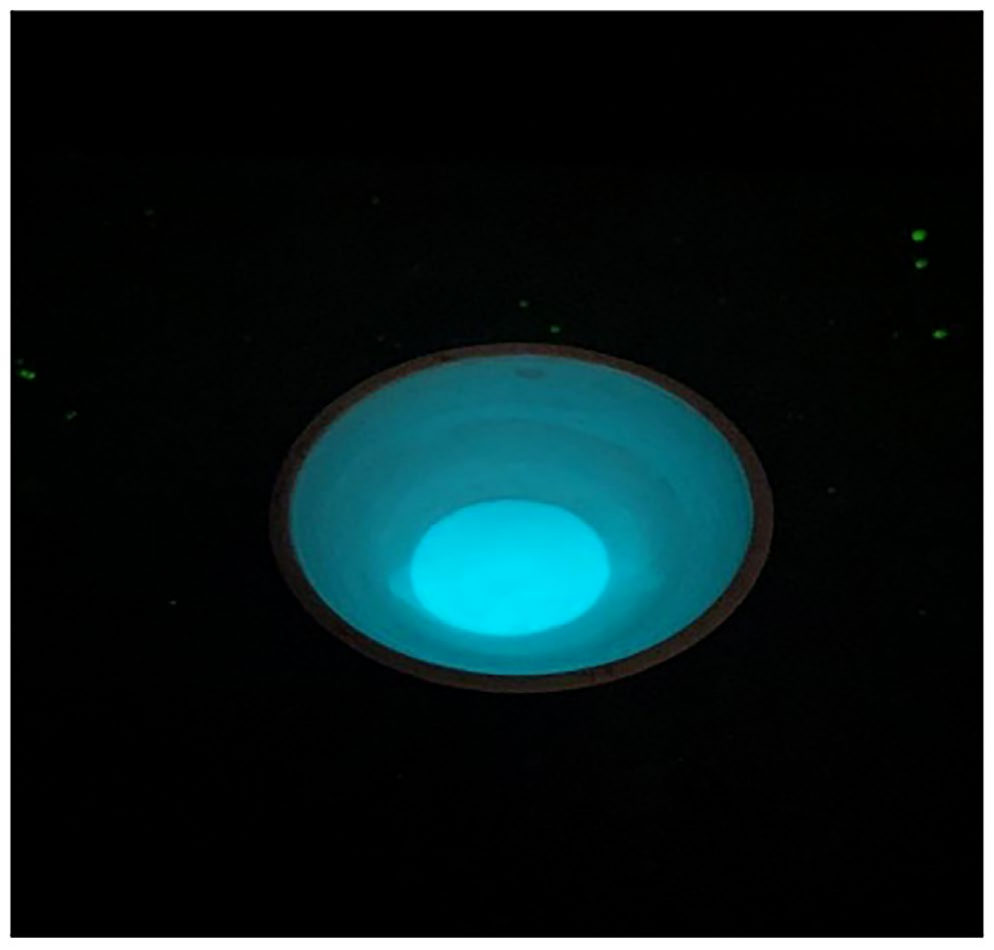
UV light examination of the sample following high‐temperature treatment (1100°C, 5‐h exposure) in a muffle furnace, showing preserved luminescence properties.

Thermal degradation analysis revealed that europium and dysprosium‐doped strontium aluminate retains its essential luminescent properties even under extended exposure to elevated temperatures. This remarkable thermal stability suggests the compound's suitability for cartridge applications, where resistance to thermal variations is essential. The effects of high‐temperature exposure (900–1300°C) over a 5‐h period are illustrated in Figure 3. The aluminate demonstrated excellent thermal stability by maintaining both its physical characteristics and luminescence intensity. These results confirm the material's capacity to not only endure high temperatures but also preserve its critical luminescent properties.

The material's ability to maintain luminescence under high‐temperature conditions indicates significant thermal resistance, which results from its stable crystalline structure incorporating europium and dysprosium dopants. These properties establish the compound as a promising option for applications involving high‐temperature exposure, such as firearm discharge environments. Quantitative results from the total solids analysis are presented in Table 1.

**TABLE 1:** Quantification of total solids content following thermal treatment at designated temperatures for 5 h in a muffle furnace.

Temperature (°C)	Sample mass (g)	Final mass (g)	Total (%)
900	5.0527	5.0460	98.87
	5.0318	5.0245	99.85
	5.0348	5.0274	99.85
		Average	99.86%
1100	5.0145	5.0104	99.92
	4.9997	4.9958	99.92
	5.0081	5.0050	99.94
		Average	99.93%
1300	5.1632	5.1647	100.03
	4.9153	4.1371	84.17
	4.9474	5.1105	103.30
		Average	95.83%

It should be noted that at 1300°C, minor variations in ash content were observed. These differences may be attributed to either slight degradation of the salt compound or inherent gravimetric measurement uncertainties. Nevertheless, these variations are not anticipated to significantly impact the material's performance as a ballistic additive [26].

### 3.3 PSD by laser diffraction

**FIGURE 4:**
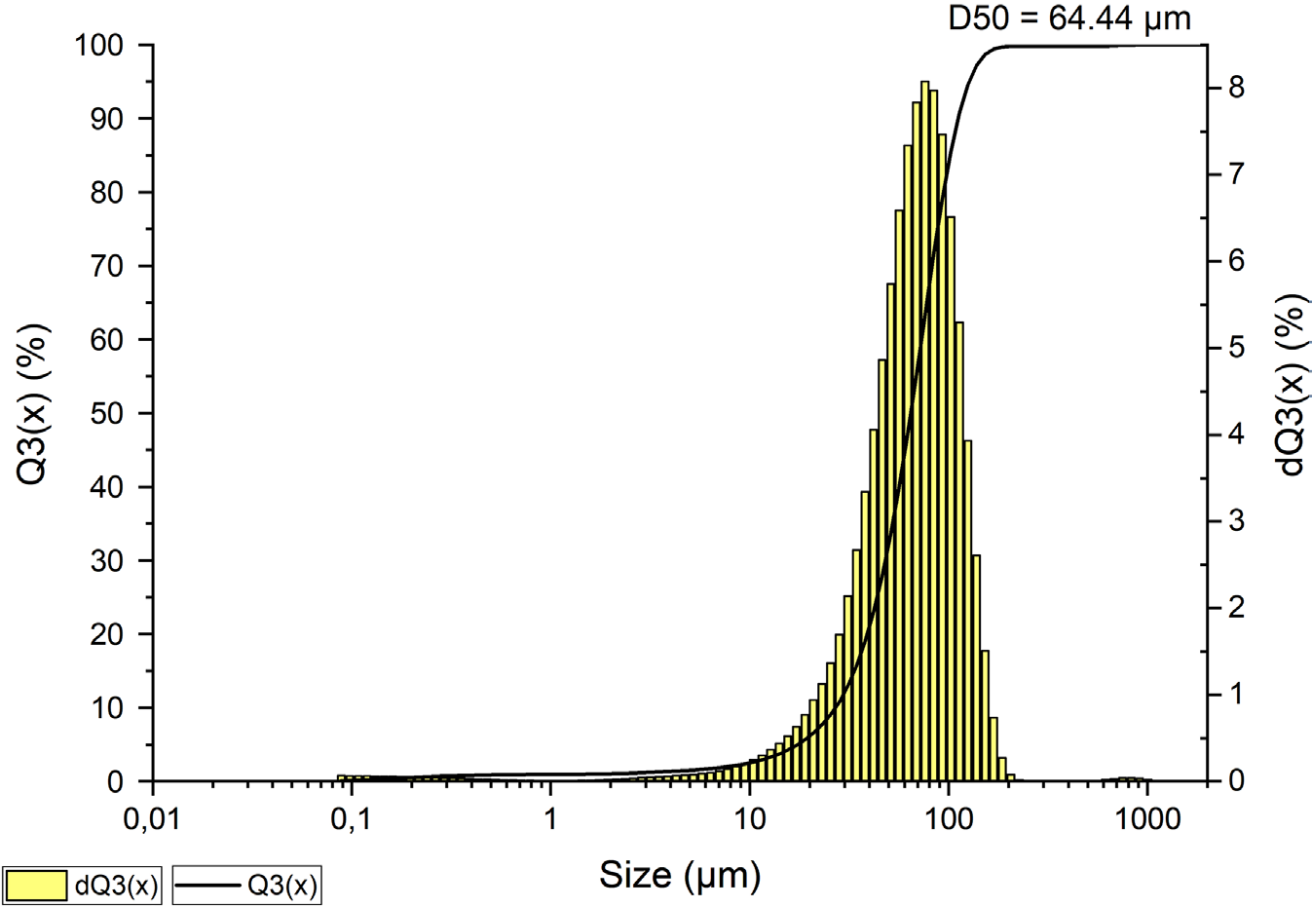
Distribution of particle sizes measured by laser diffraction spectroscopy, illustrating the primary size ranges of the analyzed salt particles. The graph highlights the most representative particle dimensions observed in the sample.

Laser diffraction analysis was conducted to quantitatively assess the PSD of the sample. The results validated the qualitative observations from SEM analysis, demonstrating a broad size distribution with particle diameters ranging from 17.50 to 158.94 μm. The complete distribution profile is illustrated in Figure 4.

The PSD analysis yielded characteristic values: D10 of 26.92 μm (indicating the diameter at or below which 10% of particles are found), D90 of 112.38 μm (representing the diameter at or below which 90% of particles are found), and a median diameter (D50) of 64.44 μm. This size distribution can be attributed to specific material processing parameters, particularly milling procedures and synthesis temperature conditions.

These results are consistent with previous research findings. Rojas‐Hernandez et al. [32] employed laser diffraction to analyze the same compound, reporting a distribution range characterized by D10 and D90 values of 20 μm and 62 μm, respectively. Notably, they demonstrated that additional milling processes could effectively reduce the average particle size to approximately 0.5 μm.

### 3.4 Surface morphology and elemental mapping via SEM‐EDS

**FIGURE 5:**
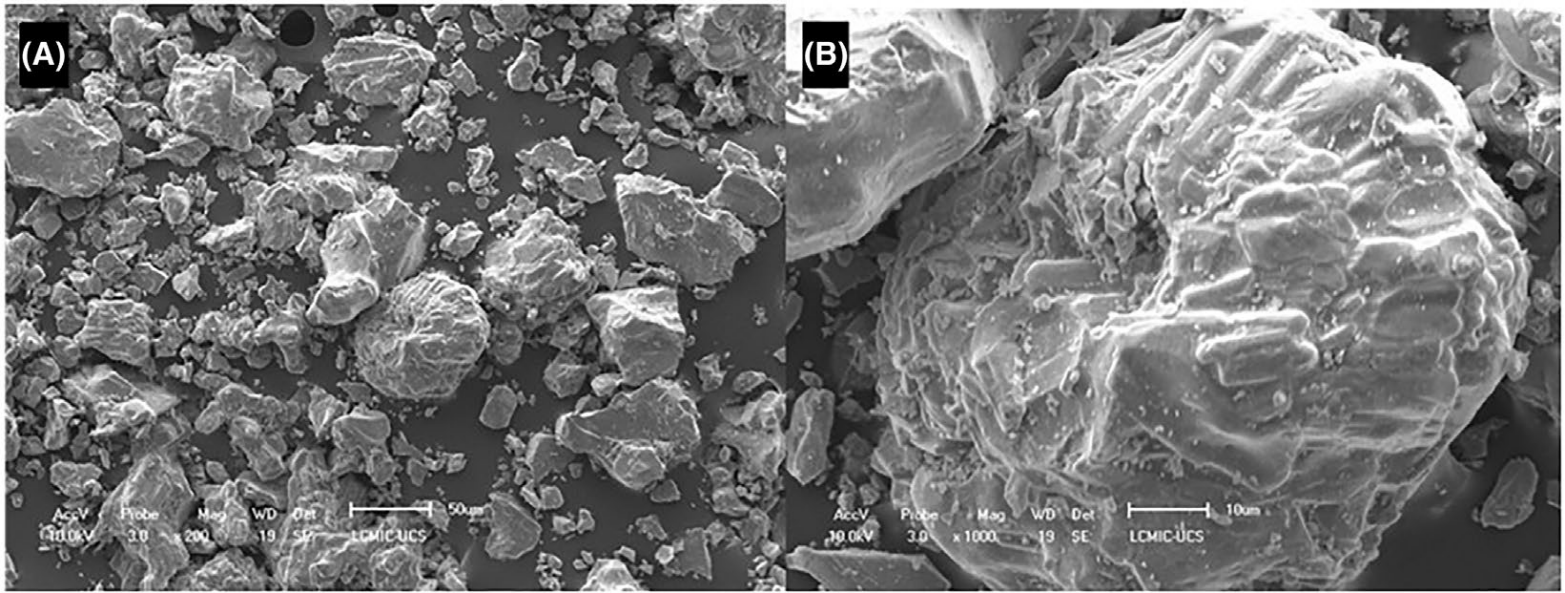
Scanning electron microscopy analysis of strontium aluminate powder. (A) Overview of particle morphology and size distribution at 200× magnification. (B) Detailed examination of particle surface features at 1000× magnification of the region shown in A.

SEM‐EDS analysis was initially performed to characterize the particle morphology of the luminescent marker. Figure 5A presents a micrograph acquired at 200× magnification with a 19 mm working distance. Although the material was previously dried, the powder still displayed a polydisperse distribution with particles predominantly smaller than 100 μm. The sample exhibited irregular and fractured surfaces, with rough textures and angular features consistent with mechanical processing. The presence of broken edges and heterogeneous particle shapes suggests fragmentation during commercial production or handling, which also contributes to the broad size distribution observed. A smaller fraction of particles showed partially smooth faces, indicating localized crystalline ordering embedded within predominantly amorphous regions.

A detailed examination of an individual particle at 1000× magnification (Figure 5B) reveals pronounced surface roughness and lamellar fracture steps, characteristic of brittle mechanical breakage. These features indicate that the original particle morphology was modified during milling or manufacturing, corroborating the structural heterogeneity typically reported for commercial SrAl_2_O_4_‐based powders.

Our morphological observations align with those reported by Soares and Valerio [33], who described strontium aluminate particles displaying both well‐defined crystalline faces and amorphous regions, with hydrodynamic diameters of 68–79 nm. These findings are further supported by Pathak and Kurchania [34], who attributed the nonuniform crystal formation to temperature‐dependent disordered growth during processing, reporting mean particle sizes of 33.04 nm.

**FIGURE 6:**
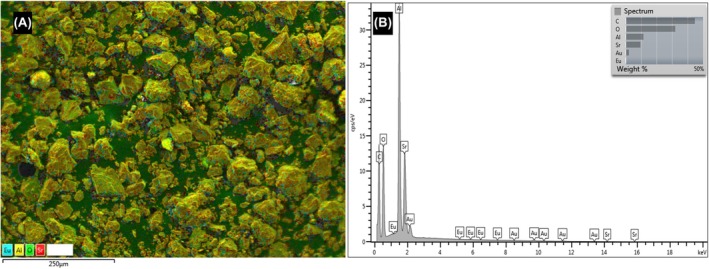
Elemental analysis of strontium aluminate by Energy‐Dispersive X‐ray Spectroscopy (EDS). (A) Elemental distribution map showing the spatial distribution of the constituent elements across the sample surface. (B) EDS spectrum displaying the relative intensities of the detected elements.

EDS analysis conducted at 75× magnification revealed the elemental composition of the material. The mapping results (Figure 6A) show predominant concentrations of oxygen and aluminum, with significant strontium content. Europium was detected in lower concentrations with heterogeneous distribution across the analyzed surface. The absence of dysprosium signals likely reflects its low concentration in the sample. The EDS spectrum (Figure 6B) confirms these elemental compositions while also showing carbon and gold signals, which can be attributed to the carbon tape substrate and gold coating used in sample preparation, respectively.

SEM analysis was also performed on the propellant grains containing the luminescent marker to verify the compound's adhesion to the powder surface. The complete set of micrographs for all formulations is provided in the Supplementary Material. Across all evaluated concentrations, the marker was visibly present on the propellant surface, with the amount of adhered particles increasing proportionally to the marker concentration.

For the formulation containing 5 wt% of the marker (Figure S1A–D), particles can be observed adhered to the surface of the propellant grains at magnifications of 500×, 1000×, 5000×, and 10,000×, indicating a consistent presence even at the lowest concentration. In the 10 wt% formulation (Figure S2A–D), a higher number of adhered particles is visible across all magnifications. The distribution remains uniform, with no indication of agglomeration or loss of surface integrity. Finally, in the 15 wt% formulation (Figure S3A–D), the surface shows an even more pronounced presence of the marker. The micrographs reveal a greater density of particles covering the powder grains, yet without compromising the morphology or structural integrity of the propellant.

EDS analyses were performed to complement the evaluation of marker adhesion to the propellant grains. For each formulation, a BSE image of the surface was acquired, followed by the generation of individual elemental maps for all elements detected in the sample, which included aluminum, strontium, oxygen, nitrogen, magnesium, and calcium, along with the corresponding EDS spectrum. The presence of these elements reflects the composition of the analyzed material. Aluminum and strontium were detected at all marker concentrations and are not typically present in single‐base propellants, which are generally composed of nitrocellulose, stabilizers, and plasticizers, without naturally occurring alkaline‐earth metals or metallic aluminum [1, 5, 35]. Therefore, the detection of these elements confirms the incorporation of the luminescent marker into the powder grains. The complete sets of BSE images, elemental maps, and spectra are provided in the Supplementary Material. Figure S4 corresponds to the formulation containing 5% marker, Figure S5 to the formulation with 10%, and Figure S6 to the formulation with 15%.

### 3.5 Structural characterization by XRD

**FIGURE 7:**
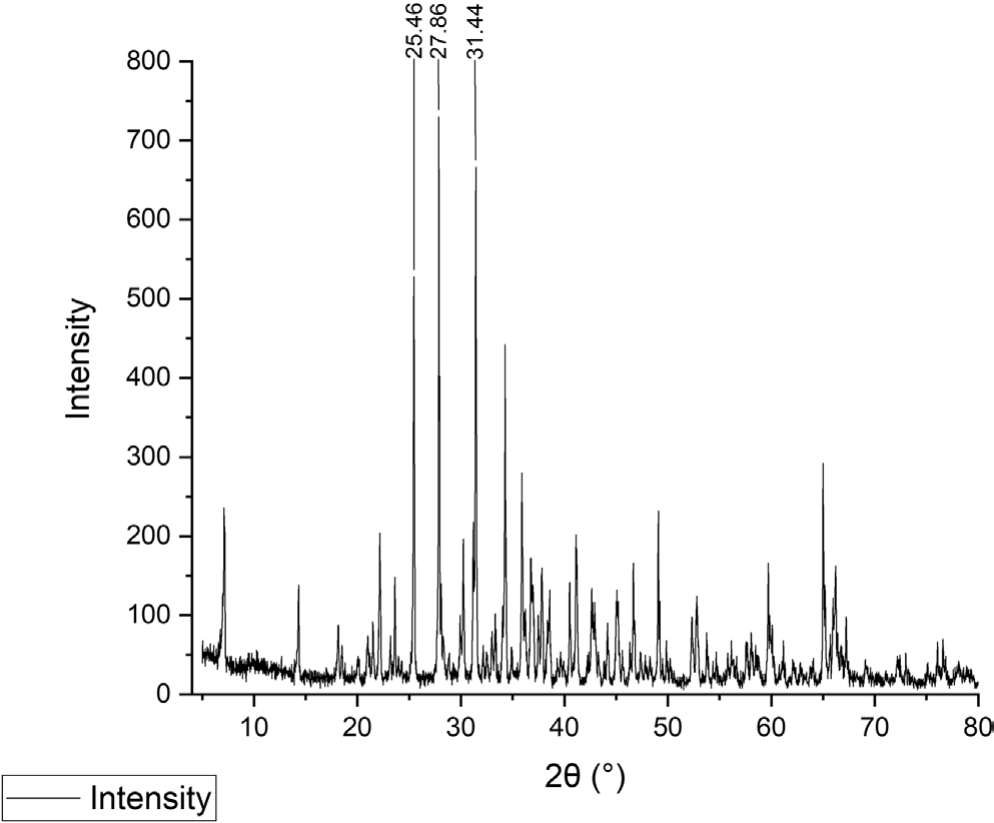
Powder X‐ray diffraction pattern of strontium aluminate. The presence of sharp, well‐defined peaks demonstrates the crystalline characteristics of the compound. Stability Assessment—Total Solids Determination.

XRD analysis was conducted to elucidate the crystal structure of the luminescent marker and validate it against existing literature data. The experimental diffraction pattern (Figure 7) revealed characteristic high‐intensity peaks at 2*θ* angles near 25° and 30°. These structural features correspond to the monoclinic phase previously documented by Soares and Valerio [33]. Additional confirmation comes from Chaware and Rewatkar [36], who reported identical diffraction patterns for the stable monoclinic structure of SrAl_2_O_4_:Eu^3+^Dy^3+^, supporting our structural characterization.

XRD analysis after the 900°C heat treatment was conducted to evaluate potential structural modifications in the doped SrAl_2_O_4_. The diffraction pattern obtained shows the same characteristic peaks previously identified in the sample before heating, maintaining the main reflections near 2*θ* ≈ 25° and 30°, which are typical of the monoclinic phase reported for SrAl₂O₄:Eu^3+^Dy^3+^ in the literature [36, 37]. Although the peaks became more intense and better defined after heating, indicating increased crystallinity, no new reflections were observed that would suggest the predominant formation of a different polymorph. Figure S7 presents the experimental diffraction pattern for the heat‐treated sample.

The literature describes that a partial transition to the hexagonal phase may occur at temperatures above approximately 650°C, especially for undoped SrAl_2_O_4_ [37]. However, in the present study, the diffraction pattern obtained after heating did not show significant changes in peak positions that would allow confirming the formation of a distinct phase. Thus, the results indicate that the thermal treatment mainly promoted recrystallization and improved structural order, while maintaining the monoclinic phase as the dominant structure. A full comparison between the two diffraction patterns is provided in the Supplementary Material, where Figure S8 shows the complete overlaid diffractograms and Figure S9 presents a zoomed view of the region between 24° and 36°.

### 3.6 Ballistics tests and UV‐light residue analysis

Ballistic evaluation was performed using reloaded cartridges incorporating the marker at various concentrations (5%, 10%, and 15% w/w), following modified protocols from Lucena et al. (2017b). Tests conducted with a Taurus® 0.38 MAG revolver (5.1″ barrel) demonstrated detectable residual luminescence from the marking agent.

**FIGURE 8:**
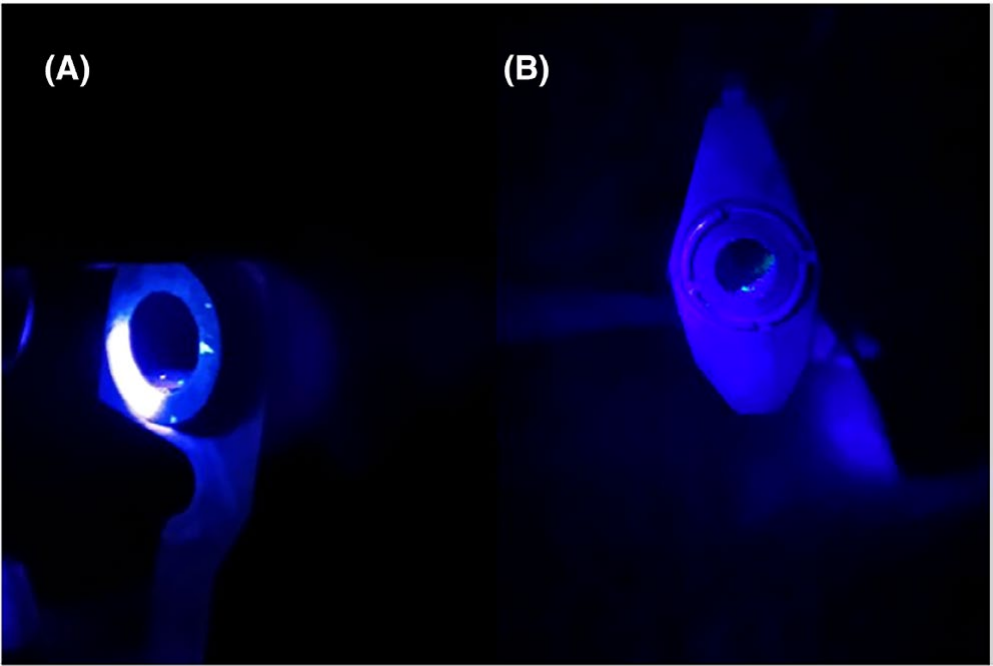
Visual analysis of marker distribution on firearm after discharge series (5% marker concentration). (A) Entrance region of barrel showing luminescence. (B) Exit region of barrel showing luminescence.

UV examination (254 nm) of the firearm revealed marker distribution on both the weapon surfaces and the shooter's hands. Initial testing at 5% concentration showed luminescence primarily at the barrel entrance and exit points (Figure 8A,B). The low concentration limited visual detection, preventing marker identification in the firing chamber and on the shooter's hands. These findings align with Weber et al. [38], who reported that while 2% mass concentration enables crime scene detection, the visibility remains minimal, consistent with our observations.

**FIGURE 9:**
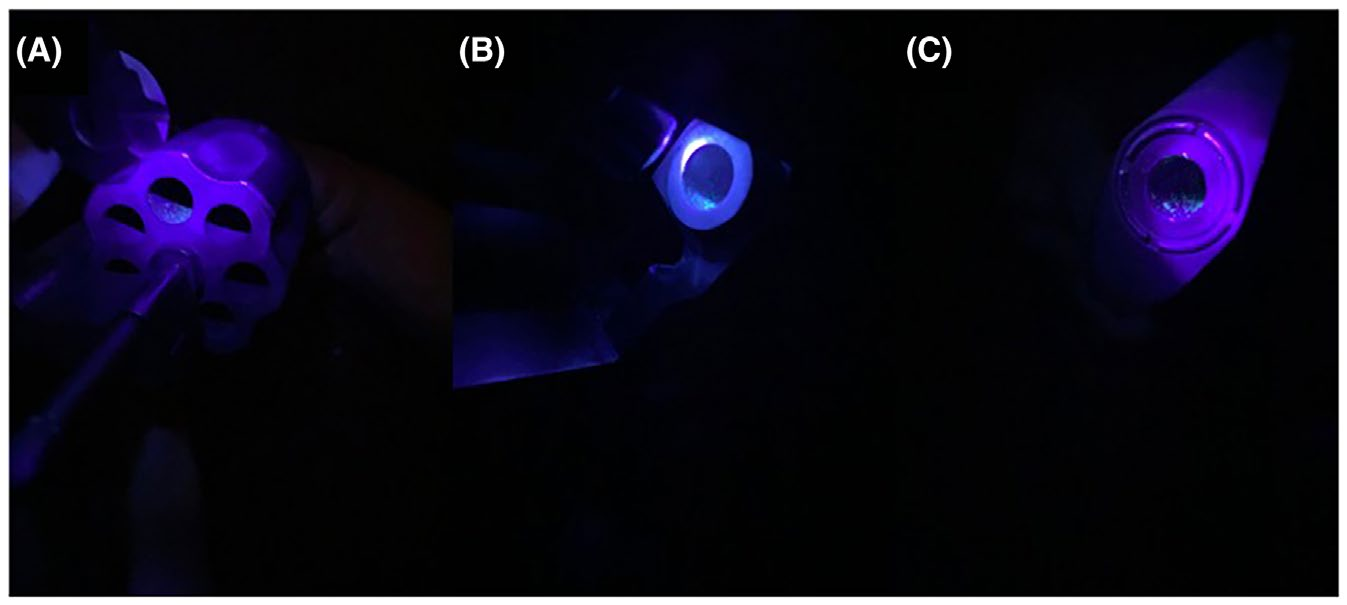
Visual analysis of the firearm after discharge series using cartridges containing 10% marker. (A) Firing chamber visualization. (B) Barrel entrance examination. (C) Barrel exit inspection.

The second experimental series, employing 10% (w/w) marker concentration, demonstrated compound detection in the firing chamber and at both barrel entrance and exit points, as shown in Figure 9A–C, respectively. This higher concentration facilitated enhanced marker detection, yielding more distinct and evident visualization compared to the previous concentration. However, luminescence remained undetectable on the firearm body and the shooter's hands.

Weber et al. [38] reported that 10% mass addition of luminescent markers achieves optimal residue visualization while maintaining acceptable projectile propulsion characteristics. Their research evaluated three novel markers at 2%, 5%, and 10% concentrations in cartridge cases. While 10% concentration produced the highest LGSR quantities, it also resulted in decreased average projectile velocity and increased misfire frequency. In contrast, 2% concentration maintained normal ballistic parameters but showed limited residue dispersion.

**FIGURE 10:**
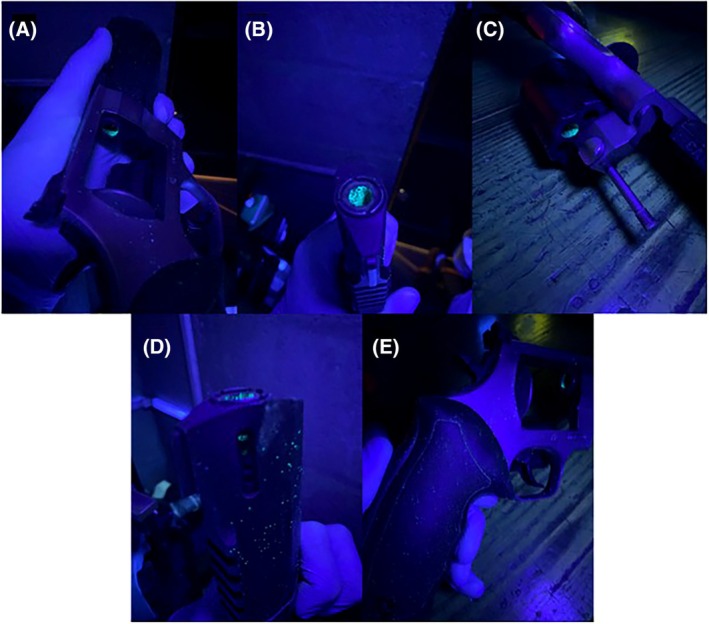
Visual analysis of marker distribution on firearm following discharge series with 15% marker concentration. (A) Barrel entrance visualization. (B) Barrel exit examination. (C) Firing chamber inspection. (D) External barrel surface analysis. (E) Firearm grip region showing marker presence.

We further evaluated a 15% mass concentration of the marker to compare its visualization efficacy with previous tests. Results indicated significantly higher compound presence, with extensive dispersion across various areas of the firearm. Marker presence was observed at the barrel entrance, firearm body, barrel exit, and accumulated in the firing chamber, as illustrated in Figure 10A through C. Unlike lower concentrations, the 15% formulation enabled compound detection on the barrel's exterior surface, as well as on the grip and frame (Figure 10C,D), demonstrating broader post‐discharge material dispersion. It is important to note that this quantity of additive did not adversely affect shot propulsion performance, and the marker's behavioral characteristics were comparable to those documented in previous research by other groups [6, 38].

**FIGURE 11:**
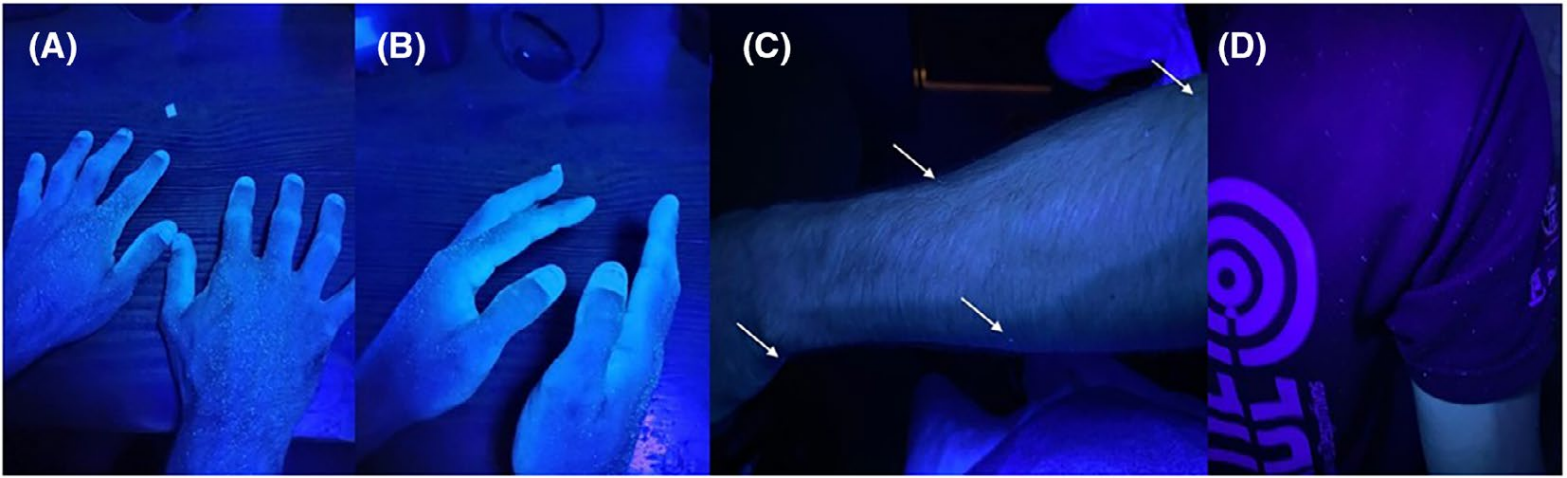
Visual analysis of marker distribution on the shooter after discharge series with 15% marker ammunition. (A) Upper surface examination of the shooter's hands. (B) Lateral view inspection of the shooter's hands. (C) Right arm region showing the marker presence. (D) Examination of the shooter's clothing.

The marker's effectiveness extended beyond the firearm, demonstrating significant adherence to the shooter's hands. Compound particles were detected specifically on the right arm and clothing worn during discharge testing, as documented in Figure 11A through D. These findings validate the marker's capacity for efficient residue transfer to surfaces near the discharge point, confirming its effectiveness as a GSR marker and supporting its potential implementation in ammunition applications.

**FIGURE 12:**
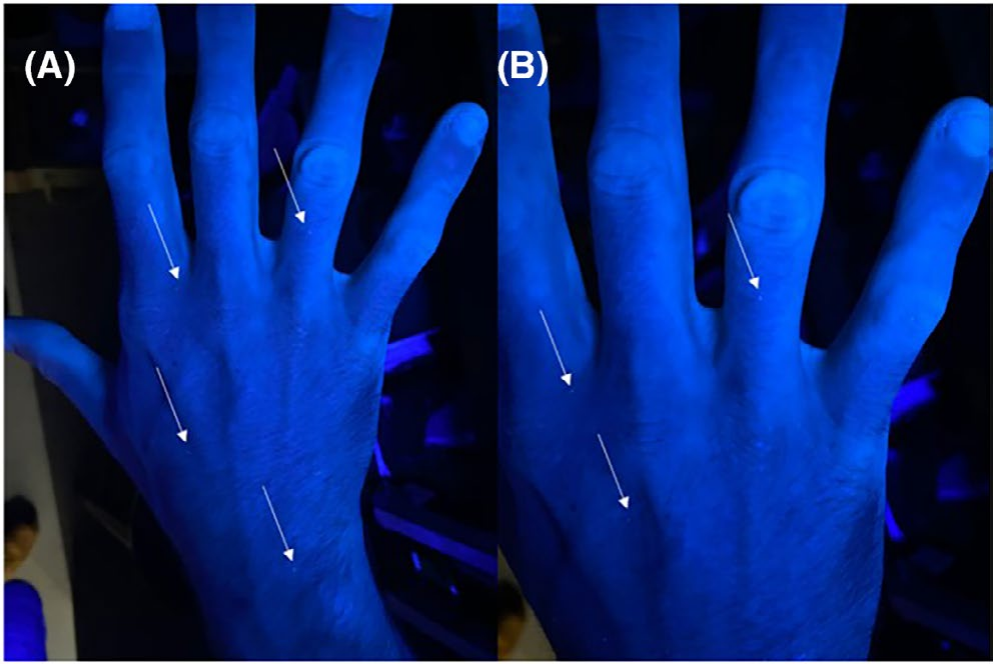
Evaluation of marker retention on shooter's hands 24 h post‐discharge. (A) Visualization of the residual marker on the shooter's hand. (B) Detailed magnification of the selected area from image A.

The persistence of marker adhesion on the shooter's hands was assessed following discharge tests using 15% compound concentration. Despite maintaining regular hygiene practices, including hand and arm washing, showering, and routine daily activities, UV examination 24 h post‐discharge revealed persistent marker presence, albeit at diminished levels. Residual particles remained detectable, as illustrated in Figure 12A, with higher magnification details shown in Figure 12B. These results confirm the marker's robust adhesion characteristics, enabling residue detection even after thorough cleaning procedures.

Previous research by Weber et al. [38] demonstrated marker detectability lasting up to 9 h, surviving 16 washing cycles. Additional literature documents that GSR can persist on shooters beyond 24 h, while GSR components on firearms and spent cartridge cases may remain detectable for periods exceeding two months [1, 5, 39].

### 3.7 Elemental analysis of post‐firing samples

**FIGURE 13:**
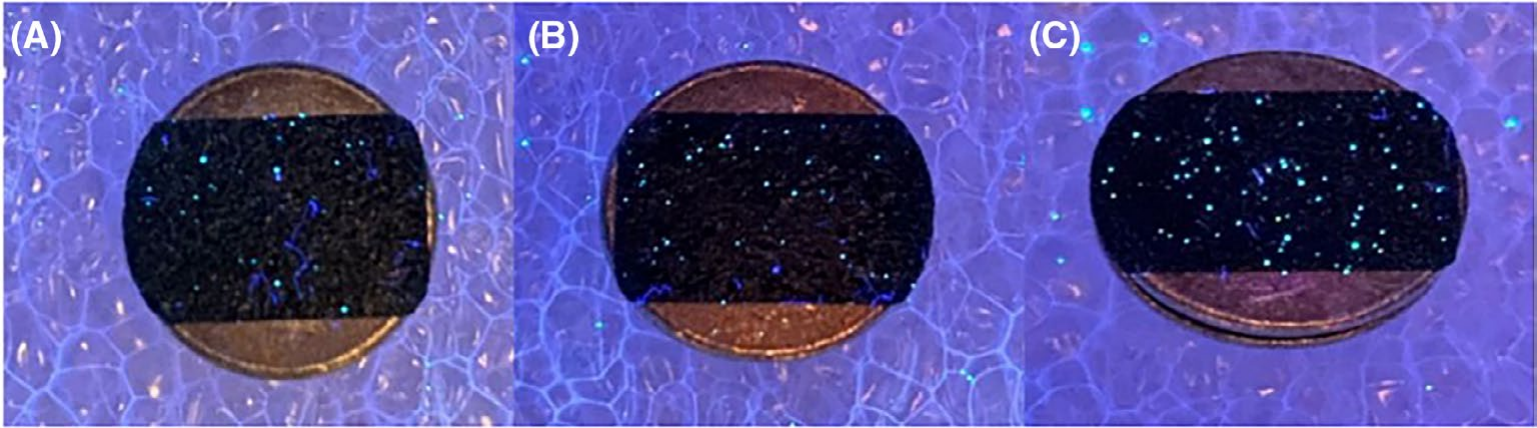
UV examination of collection stubs after sample acquisition. (A) Stub containing samples from 5% marker test; (B) 10% marker test; (C) 15% marker test.

EDS analysis was performed to validate the presence of marker compounds on the shooter's hands across all three tested concentrations, providing quantitative confirmation of visual observations. Prior to analysis, the collection stubs were examined under UV illumination to visually confirm marker presence. This preliminary screening process is illustrated in Figure 13A,B, with experiment 3 demonstrating notably higher visual intensity of marker presence, as documented in Figure 13C.

**FIGURE 14:**
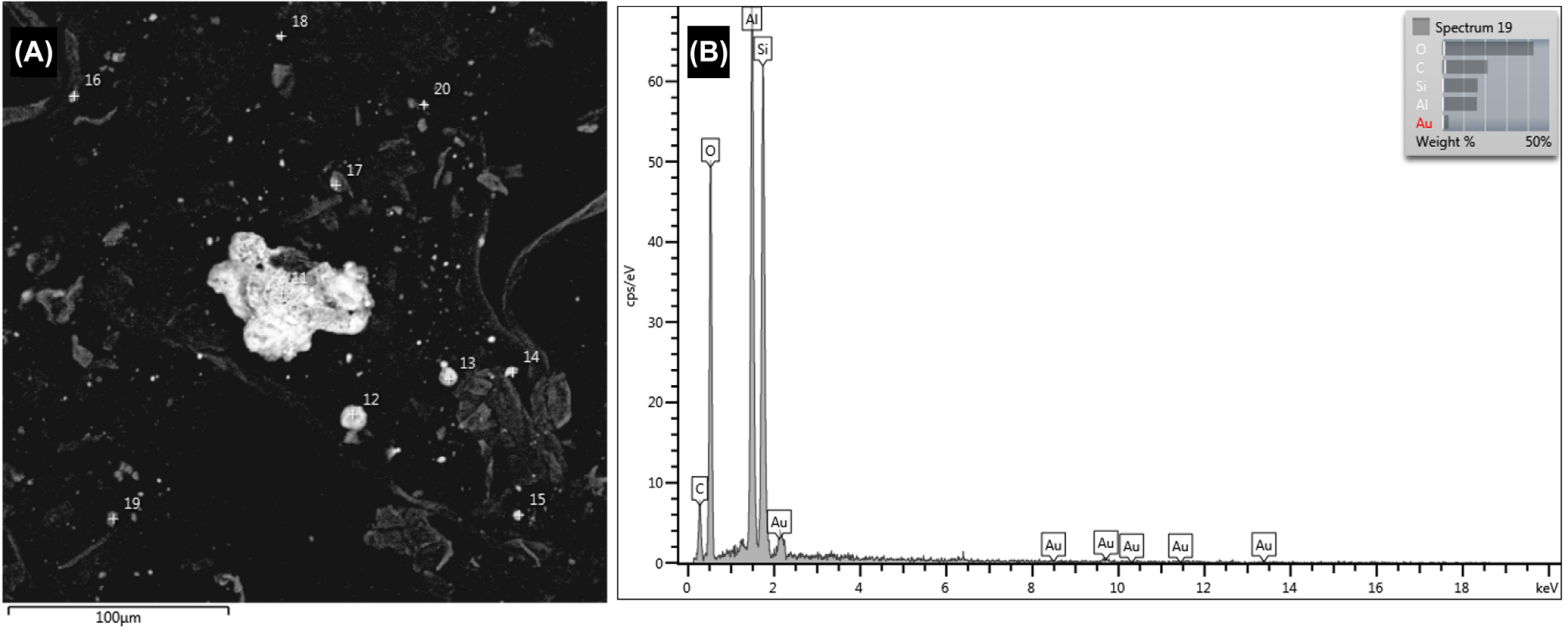
(A) Backscattered electron (BSE) micrograph of the sample from the 5% luminescent marker concentration experiment, highlighting the locations selected for EDS analysis.(B) Representative Energy‐Dispersive X‐ray Spectroscopy (EDS) spectrum of particle 19 from the 5% luminescent marker sample, showing the characteristic peaks and relative intensities of the detected elements.

The findings reinforce the relationship between marker concentration and skin retention on the shooter, with maximum detection achieved in the 15% (w/w) experiment. Subsequent elemental composition analysis by EDS was performed on all collected samples. For the 5% w/w marker concentration (experiment 1), multiple sampling points were analyzed, as shown in Figure 14A. The chemical composition analysis at point 19, shown in Figure 14B, revealed aluminum and oxygen as predominant elements, aligning with the marker's known composition. Additional signals included carbon (from the adhesive collection tape) and gold (from the conductive coating applied for analysis). A notable silicon signal was also detected. Feeney et al. [5] suggest that silicon presence typically indicates either aluminum silicate stabilizers or fuel components such as silicon monoxide and calcium silicide. The spectra obtained for the other particles are available in the Supplementary Information, Figures S10–S12.

Although such compounds can be present in GSR, the absence of calcium signals in the sample suggests that the detected silicon is unlikely to originate from calcium silicide. An important consideration is the potential misidentification between silicon and strontium signals, as their characteristic X‐ray energies are very similar (Si Kα: 1.739 keV; Sr Lα: 1.807 keV). This ambiguity arises from the inherent energy resolution limitations of EDS detection systems, which may not provide sufficient discrimination between these elements when present at low concentrations [40].

**FIGURE 15:**
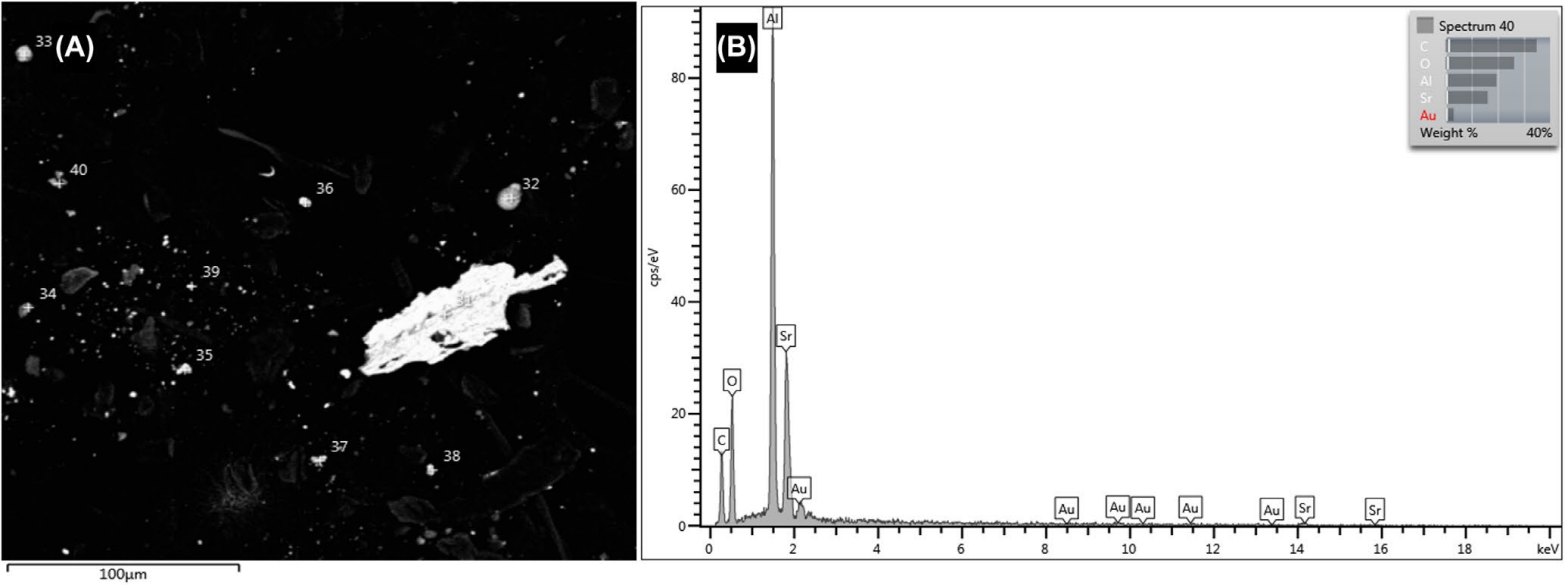
(A) Backscattered electron (BSE) micrograph of the sample from the 10% luminescent marker concentration experiment, indicating the regions selected for EDS analysis and the corresponding particle labels. (B) Representative Energy‐Dispersive X‐ray Spectroscopy (EDS) spectrum of particle 40 from the 10% luminescent marker sample, displaying the characteristic elemental peaks and their relative intensities, used to identify the chemical composition of the analyzed particle.

Figure 15A illustrates the analytical results from experiment 2 using 10% w/w marker concentration. This higher concentration produced notable changes in both signal distribution and intensity patterns, facilitating more accurate comparative analysis with the previous concentration results. Figure 15B illustrates the EDS spectral analysis of particle 40, revealing the presence of marker‐specific elements, including oxygen, aluminum, and a notably intense strontium signal. This enhanced strontium detection corresponds to the increased luminescent additive concentration, showing consistency with previously reported compositions for similar compounds [41]. However, the trace dopant elements Eu^3+^ and Dy^3+^, despite their crucial role in luminescence, were present at concentrations below the detection threshold of this analytical method.

**FIGURE 16:**
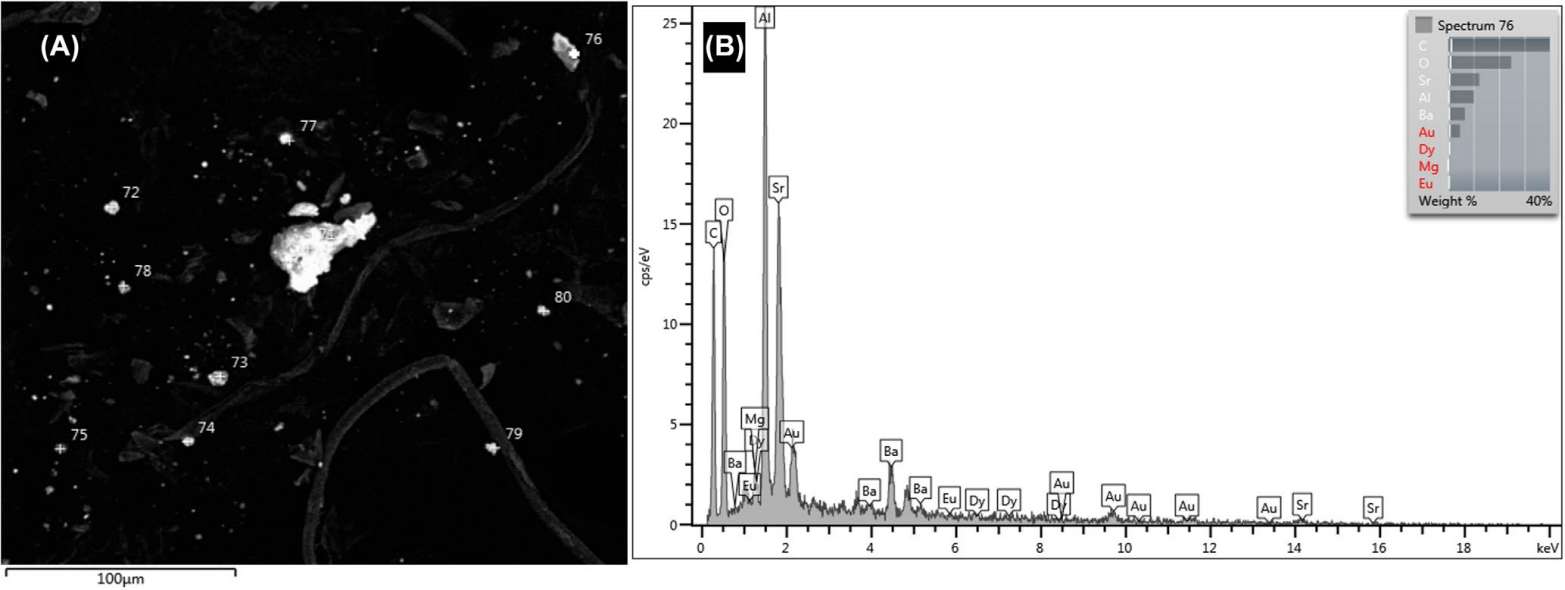
(A) Micrograph acquired in backscattered electron (BSE) mode showing the morphology and contrast of the analyzed region from the sample produced with 15% marker concentration, with the specific locations selected for EDS point analyses clearly indicated. (B) Representative energy‑dispersive X‑ray spectroscopy (EDS) spectrum corresponding to particle 76 in the 15% marker sample, highlighting the characteristic peaks used to determine its elemental composition and to correlate it with the microstructural features observed in the BSE image.

Finally, analysis of the 15% marker sample (Figure 16A) revealed significantly increased signal intensities for marker‐specific elements. The spectrum from particle 76 (Figure 16B) showed characteristic marker components including oxygen, aluminum, and strontium, with detectable levels of europium and dysprosium. Notably, barium and magnesium were also identified in the sample, elements commonly associated with conventional GSR and well‐documented in literature. The identification of these compounds suggests two possible sources of contamination: the original ammunition composition or chemical interactions between the marker and propellant mixture components. This observation highlights the critical need for further investigation into potential interactions between the marker and traditional residues. Such studies would be essential for ensuring the continued reliability of marker detection in forensic applications [5].

## 4 CONCLUSION

This study demonstrates the successful application of europium and dysprosium‐doped strontium aluminate as a luminescent marker in firearm ammunition. The compound exhibits essential characteristics for this application, including exceptional thermal stability and remarkable UV‐excited luminescence with strong surface adherence properties. SEM‐EDS analysis confirmed the presence and homogeneous distribution of marker constituent elements across surfaces exposed to discharge. UV visualization enabled efficient marker detection on the shooter's hands, barrel, and firearm frame, with persistence even after standard hygiene procedures. Among the tested concentrations (5%, 10%, and 15%), the 15% formulation demonstrated optimal performance in residue marking and tracking, providing enhanced visibility and prolonged detection capability.

The marker's structural integrity was validated through multiple analytical approaches. Visual detection using UV illumination, crystalline stability confirmation by XRD analysis, thermal resistance verification through TGA, and elemental composition confirmation via SEM‐EDS collectively established the compound's suitability for this application. These comprehensive analyses confirm the marker's stability and effectiveness under firing conditions. These findings establish europium and dysprosium‐doped strontium aluminate as a promising forensic tool for enhanced shooter identification, improved discharge location determination, more efficient gunshot residue analysis, and advanced ballistic evidence collection. This marker system represents a significant advancement in forensic science, offering new capabilities for criminal investigations involving firearms. Its implementation could substantially enhance the efficiency and reliability of gunshot residue analysis in forensic applications.

Importantly, the luminescent marker is not intended to replace conventional SEM‐EDS examination, but rather to complement it. UV‐assisted visualization can guide forensic examiners by rapidly identifying regions with higher residue accumulation, facilitating more precise and efficient sample collection for subsequent SEM‐EDS confirmation. Thus, the marker functions as an operational tool that supports and optimizes established analytical workflows, improving the overall reliability and practicality of gunshot residue investigations.

The potential implications of this research extend to standardization in ammunition manufacturing, enhanced forensic investigation protocols, improved public safety through better evidence collection, and advanced capabilities in criminal case resolution. This research provides a foundation for implementing luminescent markers in ammunition, potentially revolutionizing forensic ballistics analysis and contributing to more effective criminal investigations.

## CONFLICT OF INTEREST STATEMENT

The authors have no conflicts to declare.

## Supporting information


Figure S1.

## Data Availability

The data that support the findings of this study are available on request from the corresponding author. The data are not publicly available due to privacy or ethical restrictions.
