# Supplementary material for: Europium‐ and dysprosium‐modified strontium aluminate: A luminescent marker system for enhanced gunshot residue detection
Source: J Forensic Sci. 2026 Apr 12;71(4):1607–20. doi: 10.1111/1556-4029.70328 (PMC13340940; doi:10.1111/1556-4029.70328)
Supplement: Supplementary file 1 — Figure S1. [file JFO-71-1607-s001.docx]

**Supplemental Information**


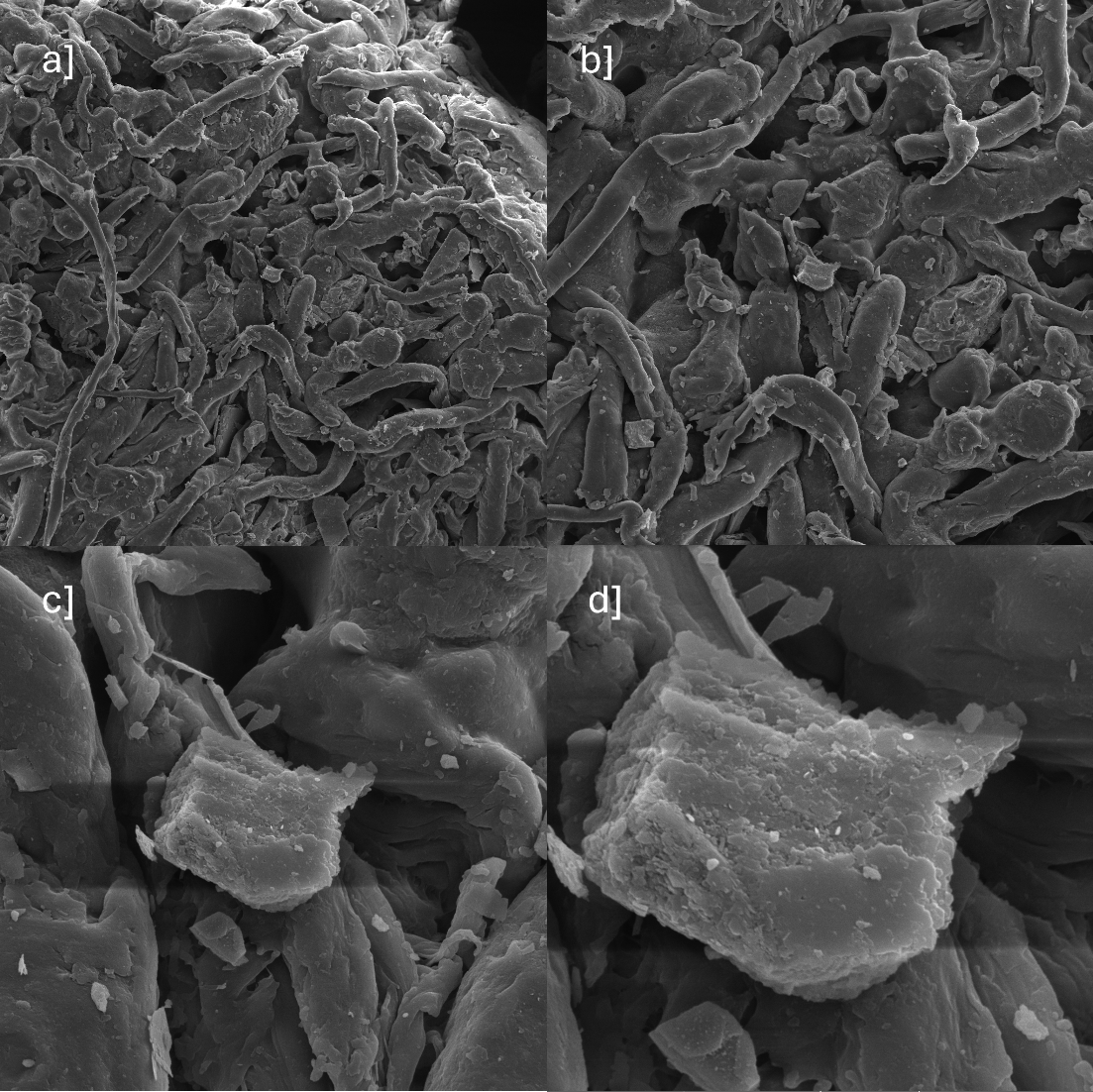


### FIGURE S1 SEM images illustrating the adhesion of the luminescent marker on propellant grain surfaces for the 5% formulation at increasing magnifications: (a) 500×, (b) 1000×, (c) 5000×, and (d) 10000×).


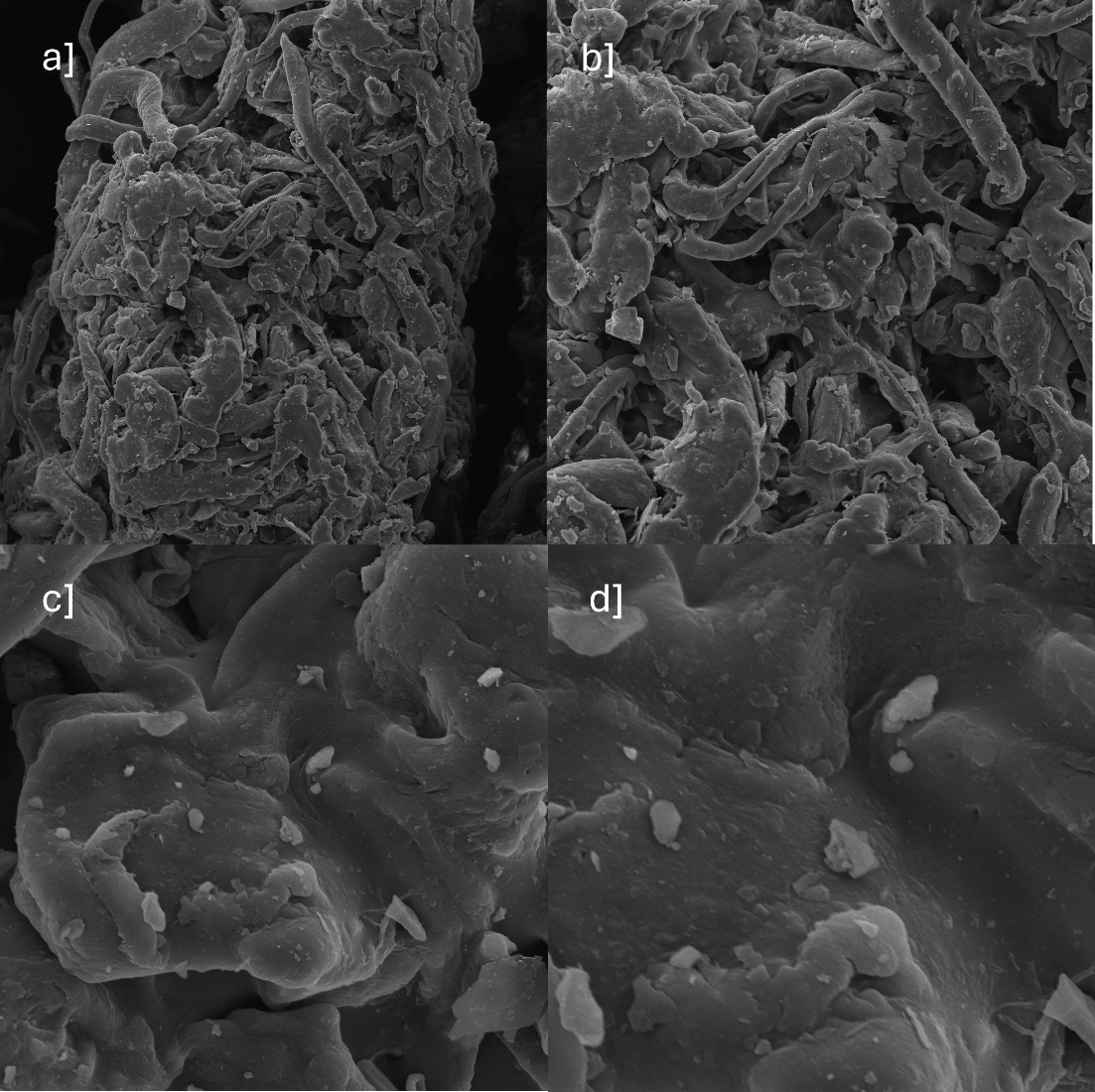


### FIGURE S2 SEM images illustrating the adhesion of the luminescent marker on propellant grain surfaces for the 10% formulation at increasing magnifications: (a) 500×, (b) 1000×, (c) 5000×, and (d) 10000×).


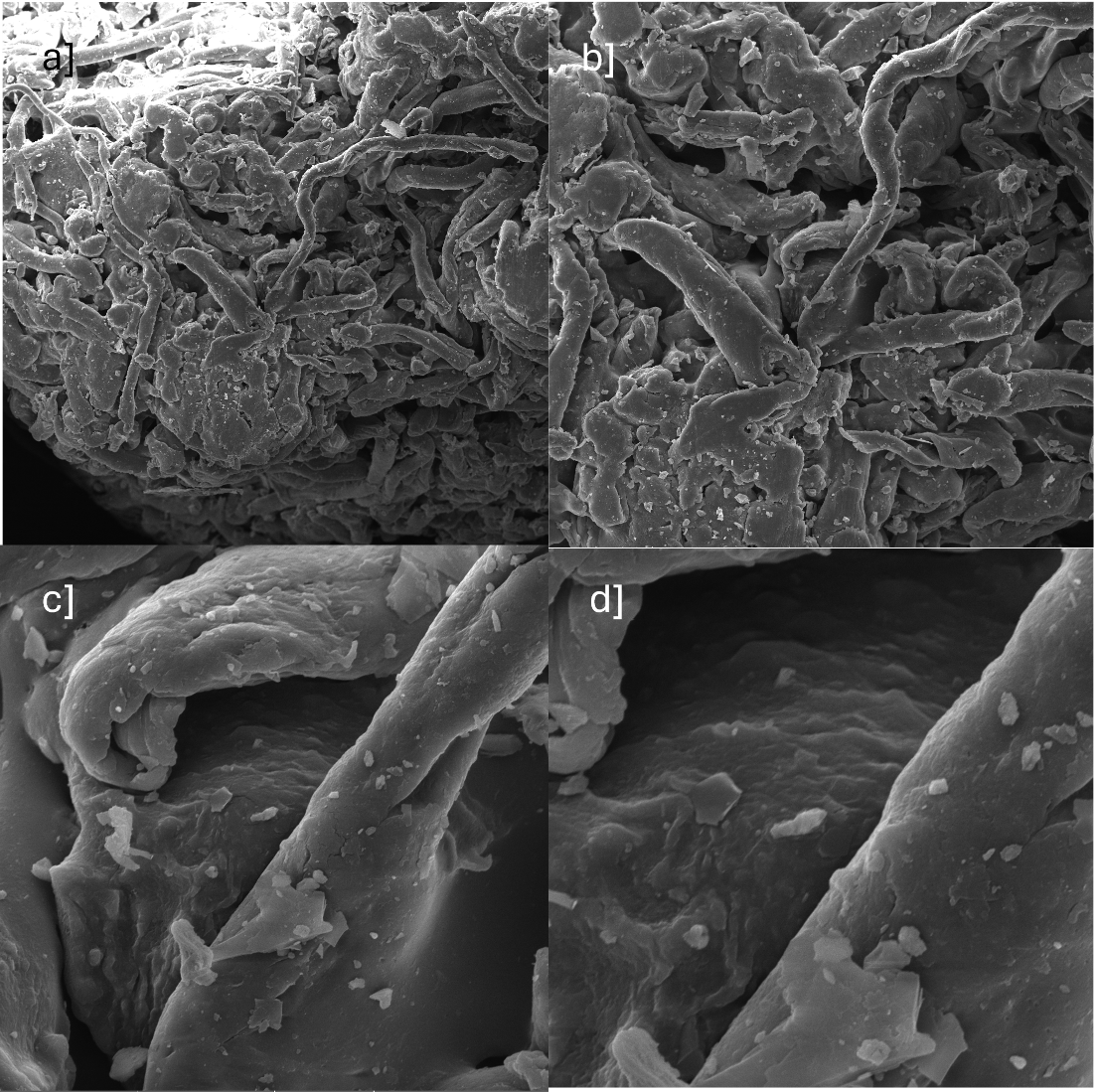


### FIGURE S3 SEM images illustrating the adhesion of the luminescent marker on propellant grain surfaces for the 15% formulation at increasing magnifications: (a) 500×, (b) 1000×, (c) 5000×, and (d) 10000×).


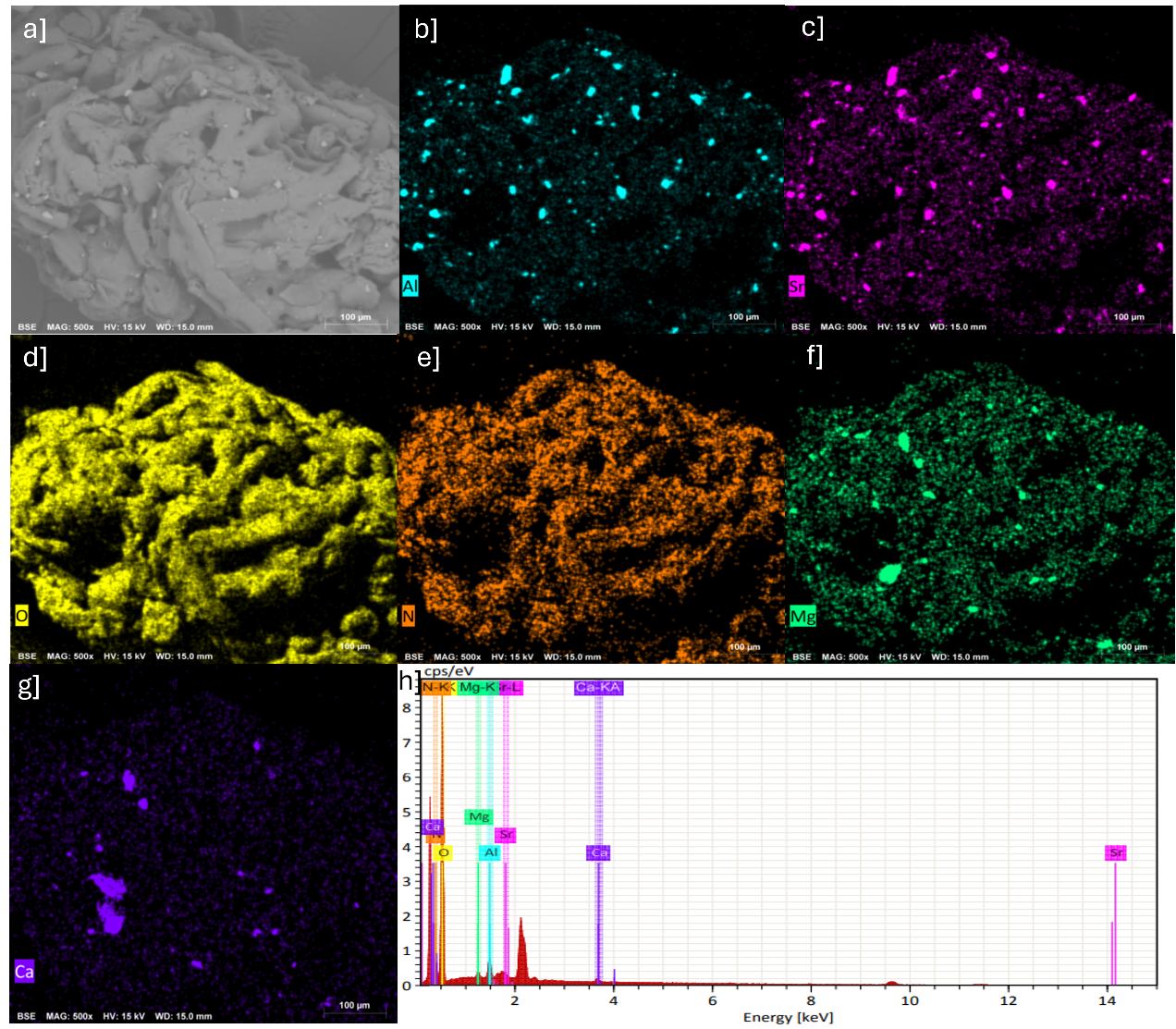


### FIGURE S4 EDS analysis showing the adhesion of the luminescent marker to the propellant grains for the 5% formulation, with the corresponding BSE image (a), elemental maps (b–g), and EDS spectrum (h).


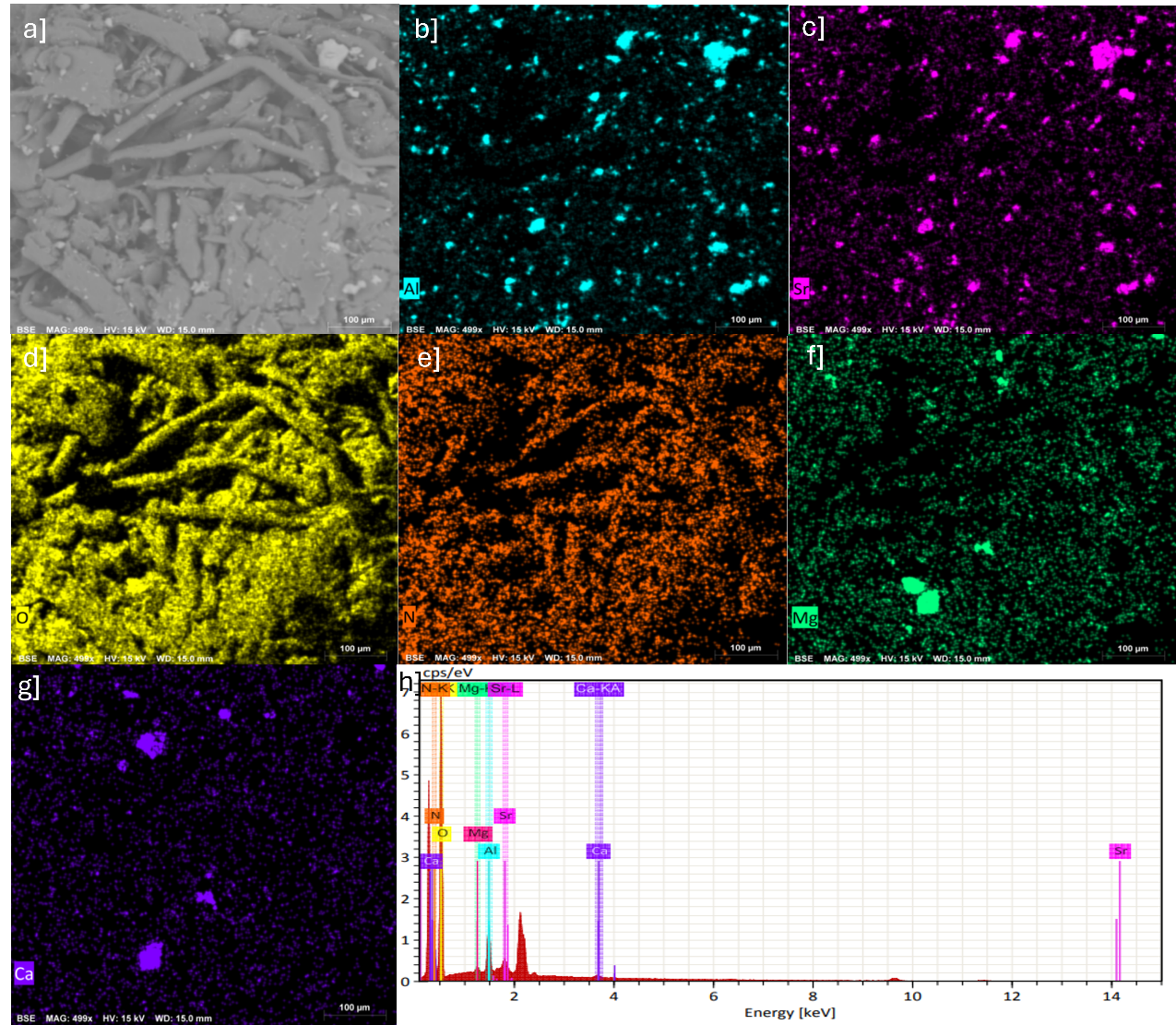


### FIGURE S5 EDS analysis showing the adhesion of the luminescent marker to the propellant grains for the 10% formulation, with the corresponding BSE image (a), elemental maps (b–g), and EDS spectrum (h).


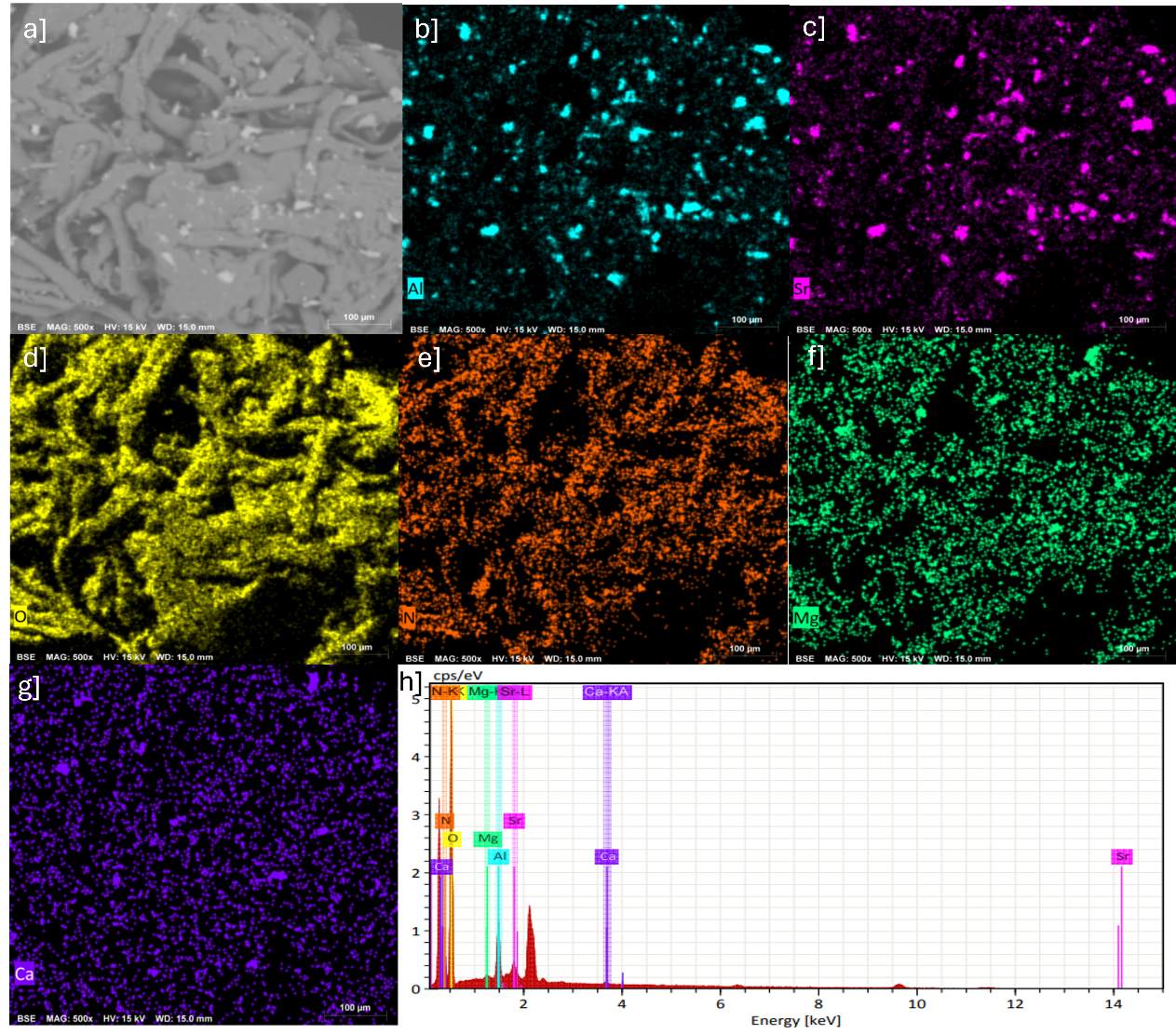


### FIGURE S6 EDS analysis showing the adhesion of the luminescent marker to the propellant grains for the 15% formulation, with the corresponding BSE image (a), elemental maps (b–g), and EDS spectrum (h).


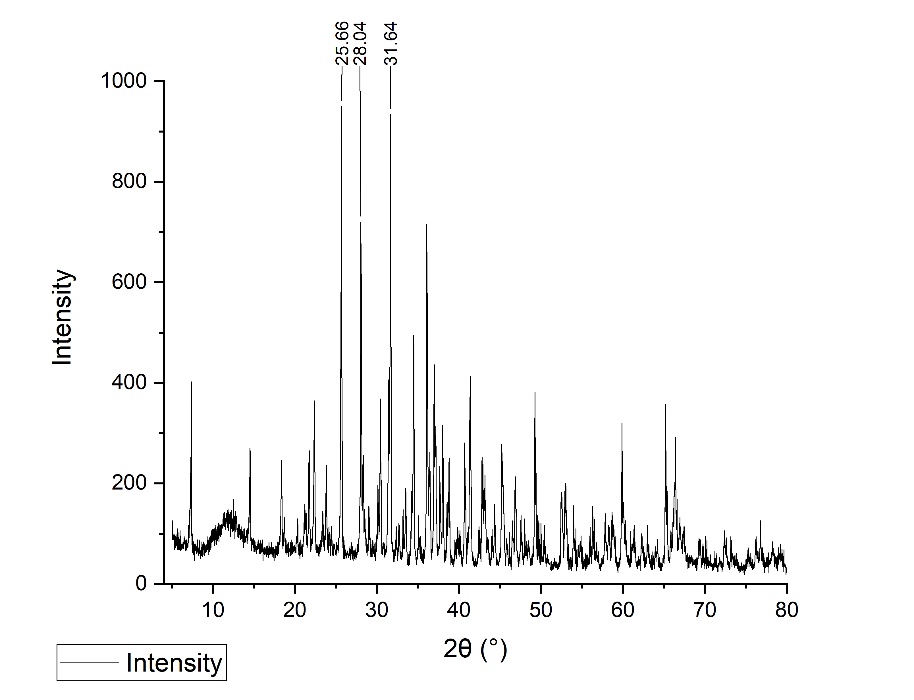


FIGURE S7 Experimental X-ray diffraction pattern of the SrAl₂O₄:Eu³⁺Dy³⁺ sample after the 900 °C heat treatment.


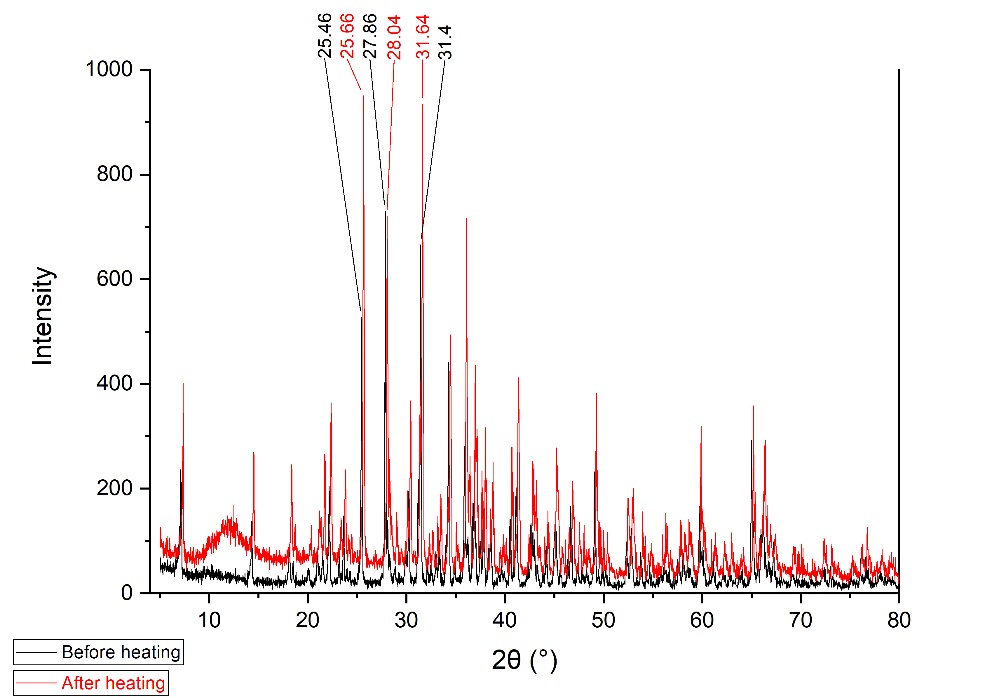


FIGURE S8 Experimental X-ray diffraction pattern of the SrAl₂O₄:Eu³⁺Dy³⁺ sample after the 900 °C heat treatment.


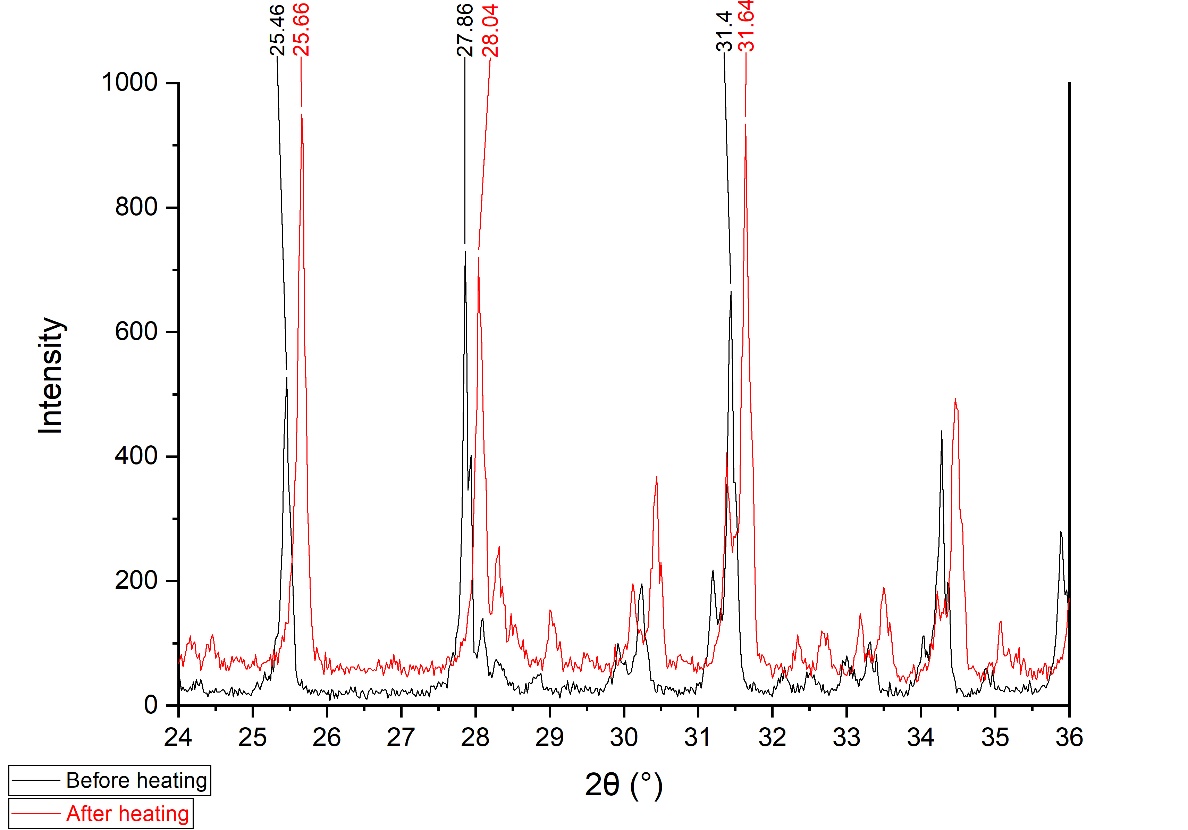


FIGURE S9 Experimental X-ray diffraction pattern of the SrAl₂O₄:Eu³⁺Dy³⁺ sample after the 900 °C heat treatment.


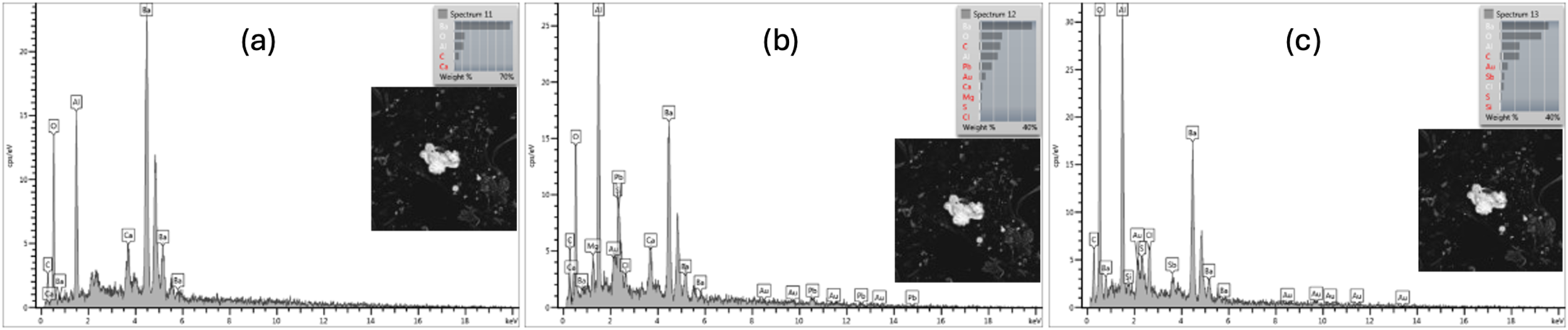


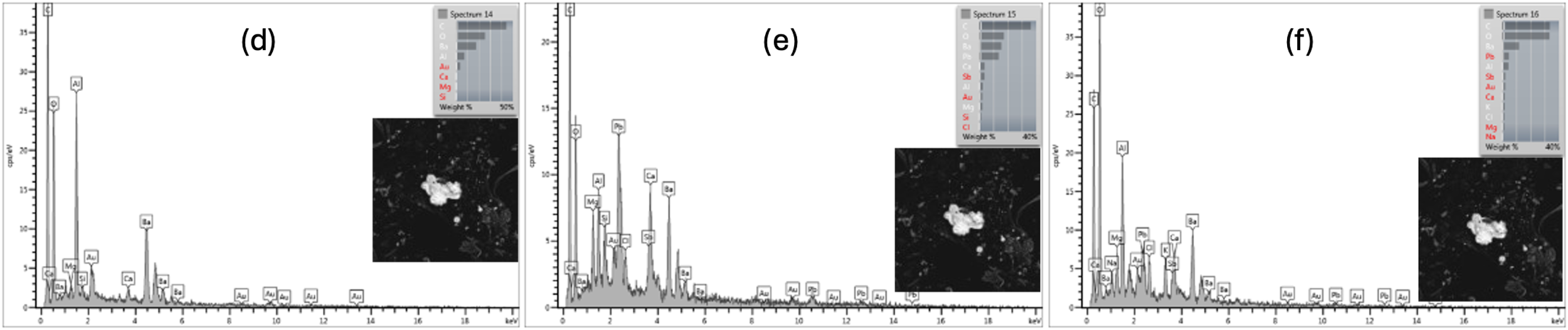


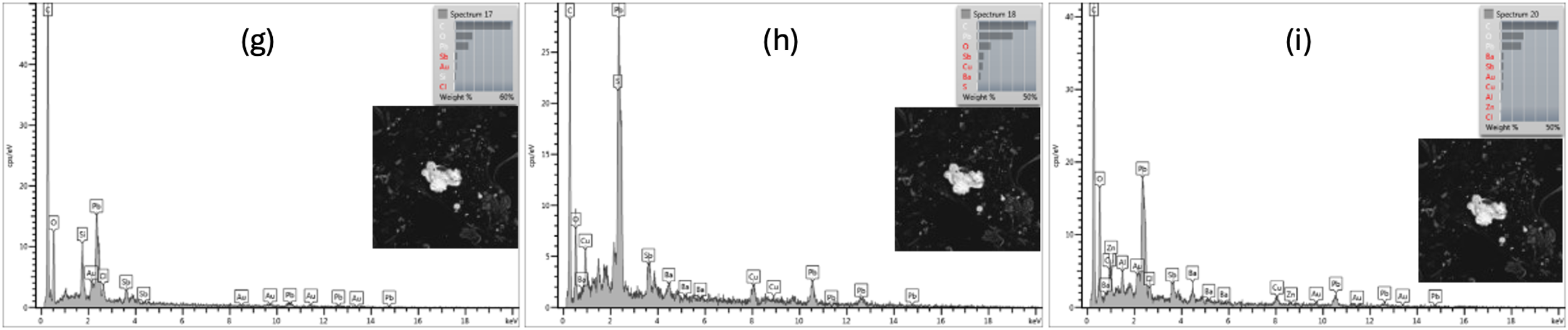


### FIGURE S10 EDS spectra obtained for particles 11 (a), 12 (b), 13 (c), 14 (d), 15 (e), 16 (f), 17 (g), 18 (h), and 20 (i), along with their micrograph for the 5% concentration.


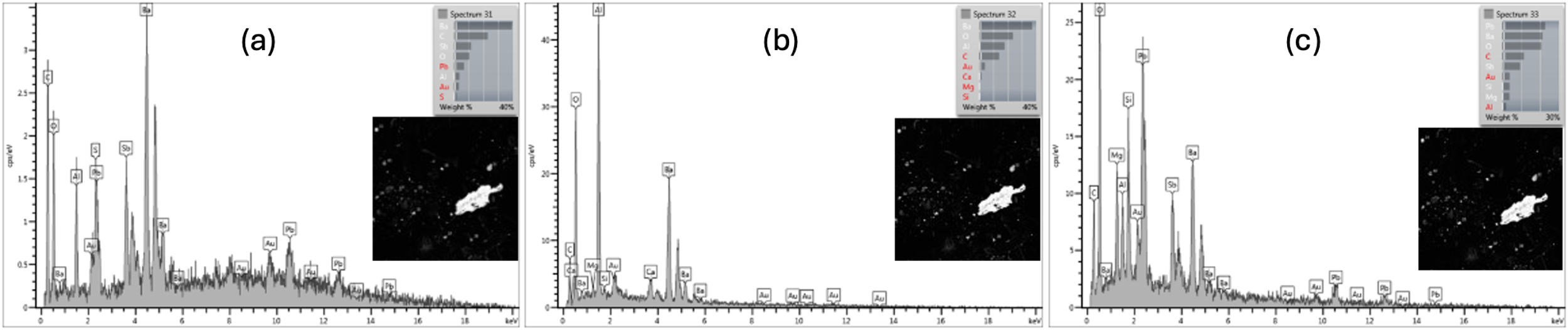


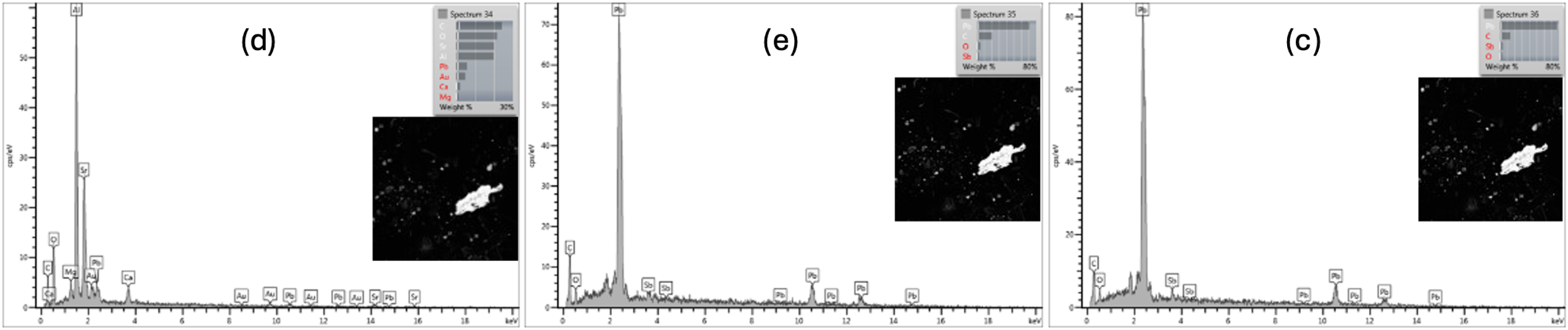


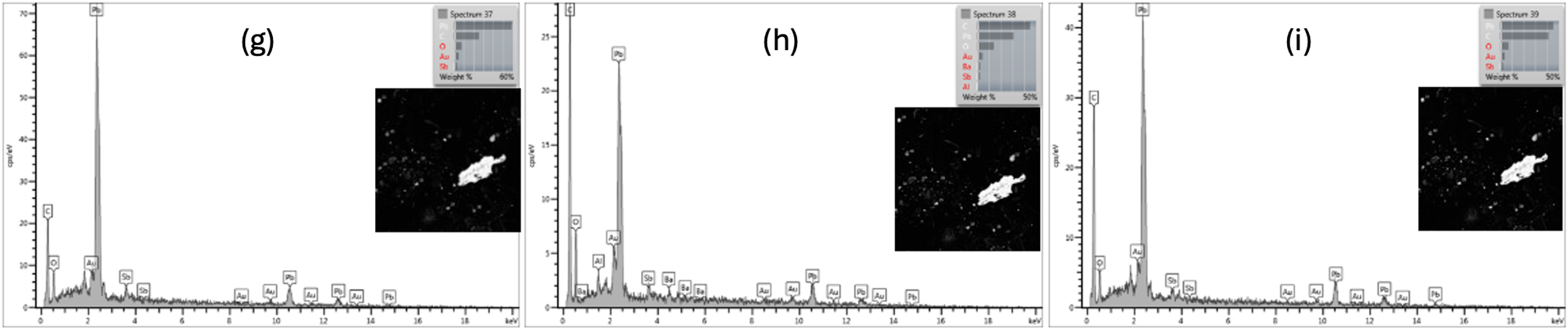


### FIGURE S11 EDS spectra obtained for particles 31 (a), 32 (b), 33 (c), 34 (d), 35 (e), 36 (f), 37 (g), 38 (h), and 39 (i), along with their micrograph for the 10% concentration.


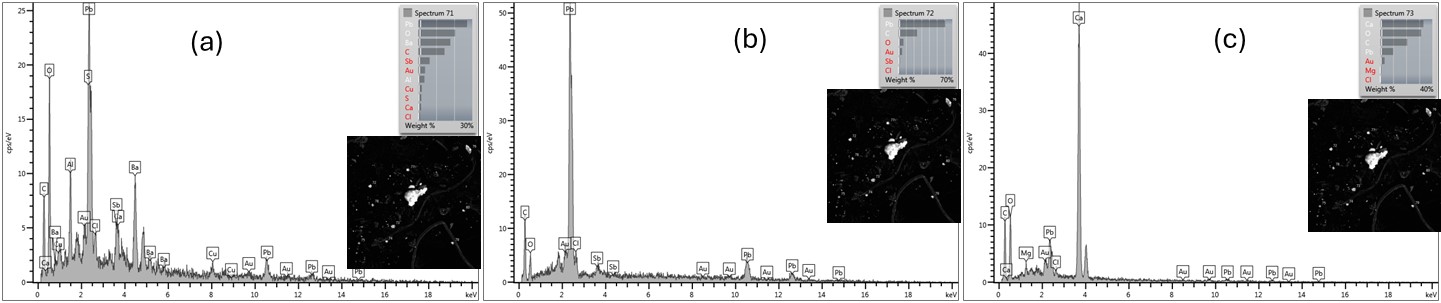


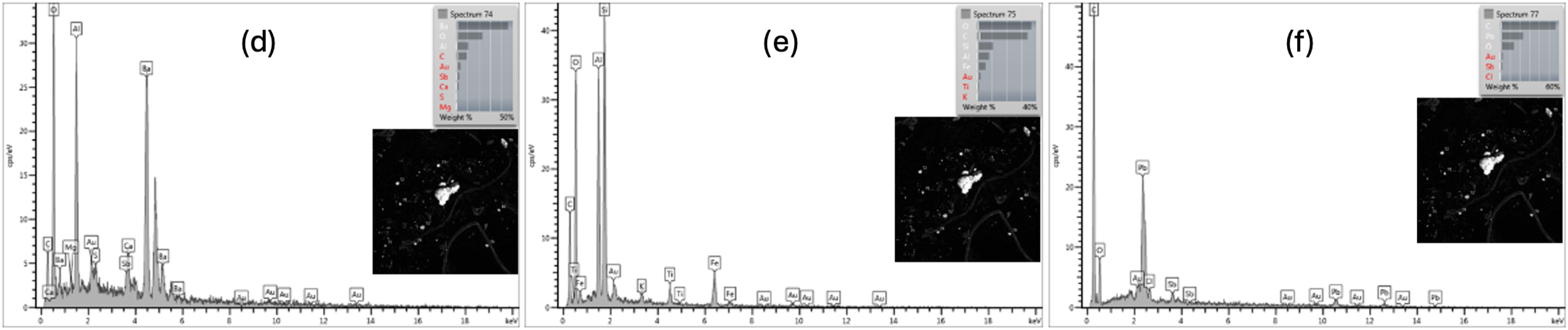


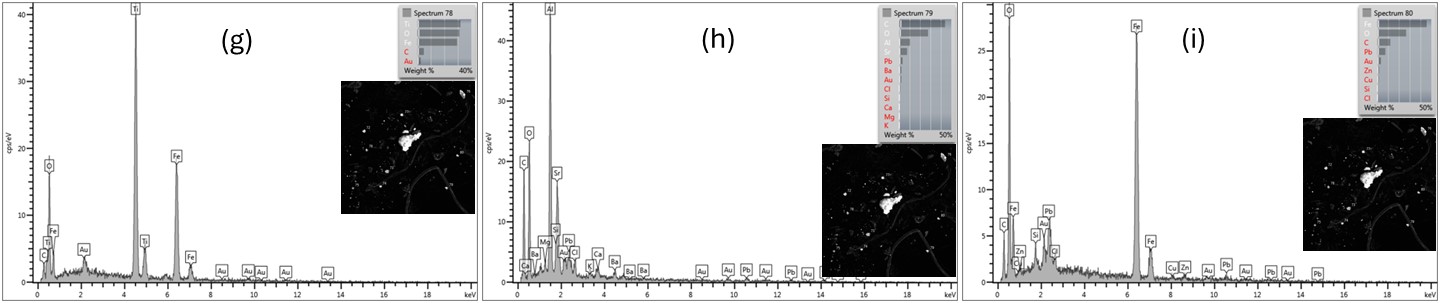


FIGURE S12 EDS spectra obtained for particles 71 (a), 72 (b), 73 (c), 74 (d), 75 (e), 77 (f), 78 (g), 79 (h), and 80 (i), along with their micrograph for the 15% concentration.
